# SNORA14A inhibits hepatoblastoma cell proliferation by regulating SDHB-mediated succinate metabolism

**DOI:** 10.1038/s41420-023-01325-0

**Published:** 2023-01-30

**Authors:** Jiabei Zhu, Siwei Mao, Ni Zhen, Guoqing Zhu, Zhixuan Bian, Yi Xie, Xiaochen Tang, Miao Ding, Han Wu, Ji Ma, Yizhun Zhu, Fenyong Sun, Qiuhui Pan

**Affiliations:** 1 Department of Laboratory Medicine, Shanghai Children’s Medical Center, School of Medicine, Shanghai Jiao Tong University, Shanghai, 200127 China; 2 Shanghai Key Laboratory of Clinical Molecular Diagnostics for Paediatrics, Shanghai, 200127 China; 3 State Key Laboratory of Quality Research in Chinese Medicine and School of Pharmacy, Macau University of Science and Technology, Macau, 999078 China; 4 Department of Laboratory Medicine, Shanghai Tenth People’s Hospital of Tongji University, Shanghai, 200072 China; 5 Sanya Women and Children’s Hospital Managed by Shanghai Children’s Medical Center, Sanya, 572000 China

**Keywords:** Cancer metabolism, Cell signalling, Small RNAs

## Abstract

Hepatoblastoma (HB) is the most common paediatric liver malignancy. Dysregulation of small nucleolar RNAs (snoRNAs) is a critical inducer of tumour initiation and progression. However, the association between snoRNAs and HB remains unknown. Here, we conducted snoRNA expression profiling in HB by snoRNA sequencing and identified a decreased level of SNORA14A, a box H/ACA snoRNA, in HB tissues. Low expression of SNORA14A was correlated with PRETEXT stage and metastasis in patients. Functionally, overexpression of SNORA14A suppressed HB cell proliferation and triggered cell apoptosis and G2/M phase arrest. Mechanistically, SNORA14A overexpression promoted the processing and maturation of the 18 S ribosomal RNA (rRNA) precursor to increase succinate dehydrogenase subunit B (SDHB) protein levels. In accordance with SNORA14A downregulation, SDHB protein expression was significantly reduced in HB tissues and cells, accompanied by abnormal accumulation of succinate. Overexpression of SDHB showed antiproliferative and proapoptotic effects and the capacity to induce G2/M phase arrest, while succinate dose-dependently stimulated HB cell growth. Furthermore, the inhibition of SNORA14A in HB malignant phenotypes was mediated by SDHB upregulation-induced reduction of cellular succinate levels. Therefore, the SNORA14A/18 S rRNA/SDHB axis suppresses HB progression by preventing cellular accumulation of the oncometabolite succinate and provides promising prognostic biomarkers and novel therapeutic targets for HB.

## Introduction

Hepatoblastoma (HB) is a primary liver malignancy arising from hepatic stem cells with an annual incidence of 1.1 cases per 1 million people in China [1]. It frequently occurs in children <5 years old, accounting for 2/3 of paediatric liver malignancies [2]. Hepatectomy, neoadjuvant chemotherapy, and liver transplantation are mainstream treatments for HB, especially early-stage HB [3, 4]. However, due to the insidious onset and difficulty in diagnosing HB early, some patients are diagnosed at an advanced stage, at which point tumours are unresectable, insensitive to chemotherapy and present worse clinical outcomes [5, 6]. Presently, only a limited number of biomarkers are available for HB diagnosis and prognosis evaluation. The serum AFP level is the most commonly used biomarker for HB diagnosis, malignant degree evaluation, therapy efficacy monitoring, and recurrence/metastasis prediction because it is significantly increased in 90% of HB patients; however, it lacks specificity for HB diagnosis because its levels are also high in normal infants and in hepatocellular carcinoma (HCC) and benign liver tumours [7–10]. Therefore, there is an urgent need to investigate the underlying mechanisms of HB pathogenesis so that sensitive and specific biomarkers for early diagnosis can be identified and novel therapeutic strategies can be developed for HB patients.

Accumulating evidence supports the driving role of metabolic reprogramming in HB initiation and development. Dysregulation of the metabolism of glucose [11], glutamine [12] and lipids [13] is implicated in providing survival advantages to HB cells. The tricarboxylic acid (TCA) cycle, as part of aerobic respiration, is central in catabolizing organic fuel molecules to supply energy for cell growth and division [14]. Succinate dehydrogenase subunit B (SDHB) is the catalytic subunit of the heterotetrametric SDH complex, which facilitates the oxidization of the TCA cycle intermediate succinate into fumarate and electron transfer from succinate to ubiquinone in the electron transport chain [15]. SDHB deficiency (in terms of expression or activity) has been found in several types of cancer and stimulates cancer progression through energy metabolism reprogramming [16], succinate accumulation [17] or an increase in reactive oxygen species (ROS) [18]. Succinate is considered an oncometabolite that promotes cancer cell proliferation, migration and invasion by activating the SKG1/KCNQ1 signalling pathway [17] and PI3K/AKT/HIF-1α signalling pathway [19] or impairing p53-driven tumour suppression [20]. However, the role of SDHB-mediated succinate metabolism in HB remains unknown.

Small nucleolar RNAs (snoRNAs) are a class of 60–300 nt noncoding RNAs (ncRNAs) that are derived from introns of host genes or transcribed from independent promoters [21]. Based on conserved elements, snoRNAs are classified into box C/D and box H/ACA snoRNAs, which canonically guide 2’-O-methylation and pseudouridylation in rRNA precursors, respectively, thus regulating rRNA maturation, ribosome biogenesis and protein translation [22–24]. Beyond their canonical roles, several box C/D snoRNAs are involved in glucose and cholesterol metabolism [25, 26]. Studies have revealed the pivotal function of snoRNAs in tumour occurrence and progression. For example, SNORA71A upregulation in lung cancer activates the MAPK/ERK signalling pathway to enhance cancer growth and metastasis [27]. SNORD76 stimulates HCC cell proliferation and migration by activating the Wnt/β-catenin signalling pathway [28]. However, the association between snoRNAs and HB has not yet been investigated. Clarification of snoRNA expression patterns and roles in HB may therefore provide new strategies for HB diagnosis and treatment.

In this study, we confirmed the reduced level of SNORA14A, a classical box H/ACA snoRNA, in HB tissues and cells, which was associated with PRETEXT stage and metastasis in patients. Mechanistic studies demonstrated that SNORA14A showed antiproliferative and proapoptotic effects and the capacity to induce G2/M arrest by upregulating SDHB protein expression and subsequently decreasing cellular succinate levels in a canonical manner dependent on promoting 18 S rRNA precursor maturation.

## Results

### Significant downregulation of SNORA14A in HB tissues and cells

**Fig. 1:**
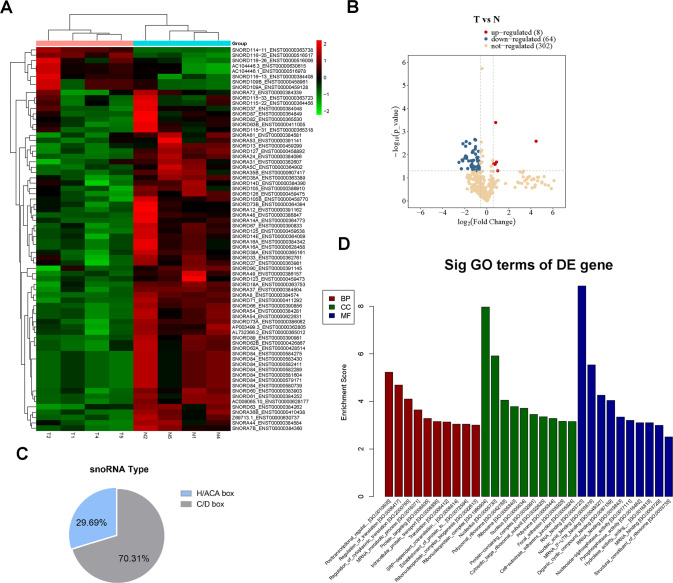
SnoRNA sequencing reveals the snoRNA expression profile of HB tissues. **A** Heatmap clustering of snoRNA sequencing results for 4 pairs of HB and NT tissues. **B** Volcano plot of significantly differentially expressed snoRNAs. **C** The types of significantly differentially expressed snoRNAs. **D** GO enrichment analysis of the host genes of significantly differentially expressed snoRNAs.

**Fig. 2:**
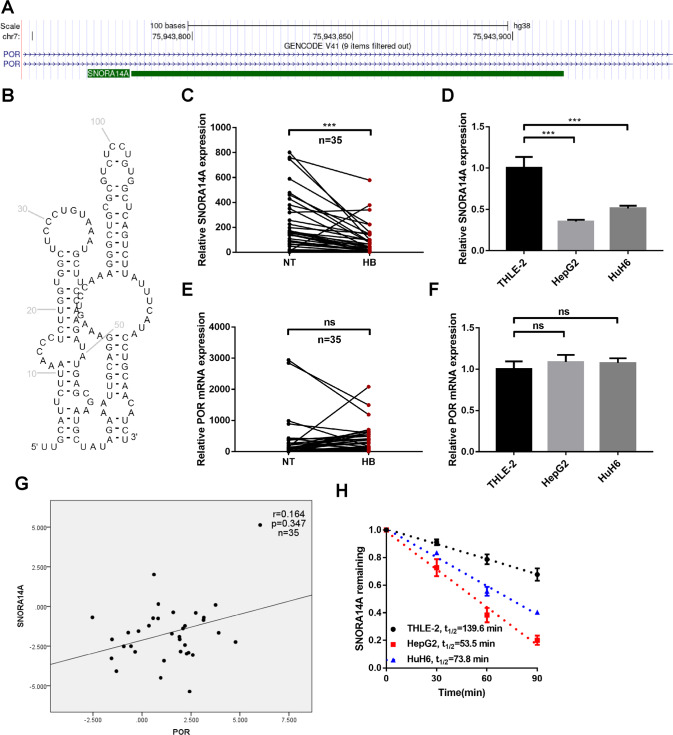
Significant downregulation of SNORA14A in HB tissues and cells. **A** The location of SNORA14A and POR from the UCSC Genome Browser. **B** The secondary structure of SNORA14A. **C**–**F** Relative expression levels of SNORA14A (**C**) and POR (**E**) in 35 pairs of HB and NT tissues were measured by qRT‒PCR. The relative expression levels of SNORA14A (**D**) and POR (**F**) in THLE-2, HepG2 and HuH6 cells were measured by qRT‒PCR. **G** Spearman rank correlation analysis of SNORA14A and POR expression levels. **H** SNORA14A levels in THLE-2, HepG2 and HuH6 cells were measured using qRT‒PCR after treatment with 5 μg/ml actinomycin D for the indicated times.

To investigate the snoRNA expression pattern in HB, we performed snoRNA sequencing in 4 pairs of HB tissues and matched adjacent nontumour (NT) liver tissues and identified 72 significantly differentially expressed snoRNAs, including 8 upregulated snoRNAs and 64 downregulated snoRNAs (Fig. 1A, B and Table S1). A total of 29.69% and 70.31% of the differentially expressed snoRNAs were box H/ACA and box C/D snoRNAs, respectively (Fig. 1C). GO enrichment analysis of host genes of differentially expressed snoRNAs confirmed that posttranscriptional regulation of gene expression, regulation of translation, and the mRNA metabolic process were enriched, suggesting that snoRNAs derived from these host genes might be involved in these biological processes (Fig. 1D). Then, we selected SNORA14A, which ranked first among 64 significantly downregulated snoRNAs according to the log2-fold change (log2 FC = −2.522), for further study. SNORA14A is generated from the first intron of the cytochrome P450 oxidoreductase gene POR (Fig. 2A). SNORA14A is 135 nt long and contains two highly conserved sequence elements, the H box (ATAGAA) and the ACA box (ACA) (Fig. 2B). qRT‒PCR was performed to determine SNORA14A expression in 35 paired HB tissues and adjacent NT tissues. We found that SNORA14A expression was markedly decreased in HB tissues compared to NT tissues (Fig. 2C). Consistently, SNORA14A expression in two HB cell lines (HepG2 and HuH6) was apparently lower than that in the human normal hepatocyte line (THLE-2) (Fig. 2D). Given that intronic SNORA14A is cotranscribed with its host gene, we measured POR mRNA levels in HB. However, POR mRNA levels did not differ between HB tissues/cells and normal liver tissues/cells (Fig. 2E, F). Spearman’s rank correlation analysis revealed no correlation between SNORA14A and POR expression levels (*r* = 0.164, *P* = 0.347) (Fig. 2G). To further investigate how the steady-state SNORA14A level was reduced in HB, we performed RNA decay assays and found that the half-life of SNORA14A in HB cells was obviously shorter than that in THLE-2 cells (Fig. 2H), suggesting that SNORA14A is less stable in HB cells than in THLE-2 cells.

### SNORA14A inhibits HB cell proliferation, promotes cell apoptosis, and arrests cells in the G2/M phase

**Fig. 3:**
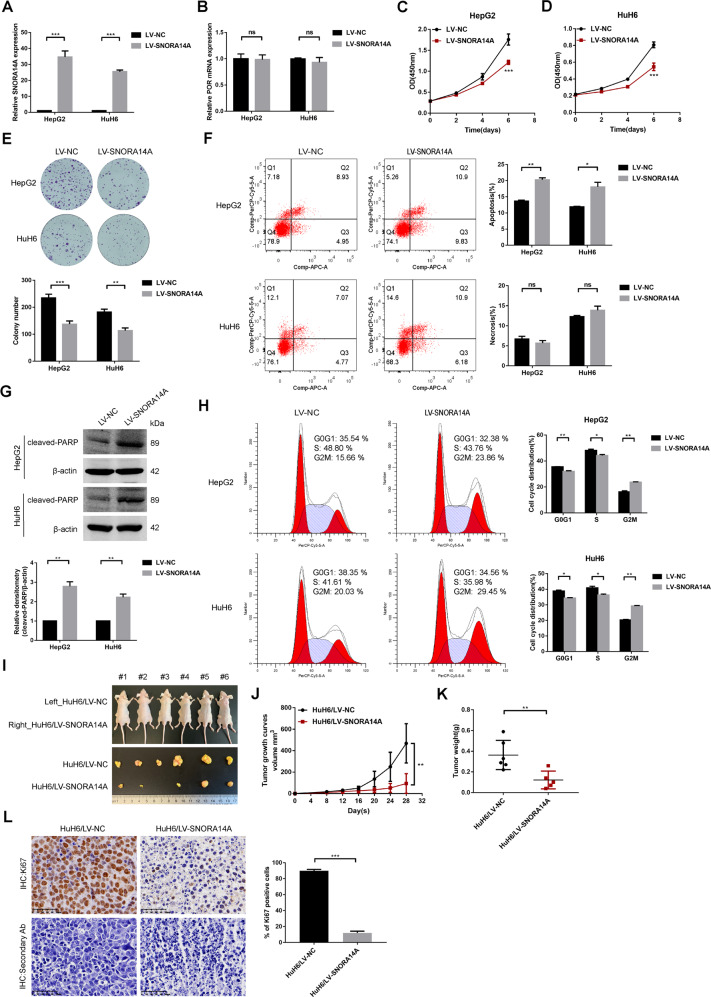
SNORA14A overexpression inhibits HB cell proliferation, promotes cell apoptosis and arrests cells in the G2/M phase. **A**, **B** The relative expression levels of SNORA14A (**A**) and POR (**B**) in HB/LV-NC and HB/LV-SNORA14A cells were measured by qRT‒PCR. **C**–**E** The proliferative activity of HB/LV-NC and HB/LV-SNORA14A cells was detected by CCK-8 (**C**, **D**) and colony formation (**E**) assays. **F** Apoptosis and necrosis of HB/LV-NC and HB/LV-SNORA14A cells were detected by flow cytometry assays. **G** Cleaved PARP protein levels in HB/LV-NC and HB/LV-SNORA14A cells were measured by Western blotting assays. Relative densitometry was performed with ImageJ. **H** The cell cycle of HB/LV-NC and HB/LV-SNORA14A cells was detected by flow cytometry assays. **I** Tumours dissected from six nude mice subcutaneously inoculated with HuH6/LV-NC (left) and HuH6/LV-SNORA14A (right) cells. **J** The tumour volumes (mm^3^) on the indicated days were calculated for tumour growth curves. **K** Average weight of dissected tumours. **L** Ki67 protein expression in dissected tumours was measured by IHC assays, in which staining with only the corresponding secondary antibody was used as a control. The percentages of Ki67-positive cells are shown.

To explore the biological role of SNORA14A in HB, we constructed HB cells stably overexpressing SNORA14A and confirmed the overexpression efficiency by qRT‒PCR (Fig. 3A). CCK-8 and colony formation assays showed that SNORA14A overexpression significantly inhibited HB cell proliferation (Fig. 3C–E). To investigate whether a mild increase in SNORA14A expression would still induce antiproliferative effects, we performed dose-dependent overexpression assays. qRT‒PCR confirmed that cells transfected with 1 μg SNORA14A overexpression plasmids showed an 11.6–16.8-fold increase in SNORA14A levels, while cells transfected with 2 μg plasmids showed a 22.7–33.8-fold increase in SNORA14A levels (Fig. S1A). Cells with different levels of SNORA14A overexpression exhibited apparently weaker proliferative activity than control cells (Fig. S1B–D). Flow cytometry and Western blotting experiments demonstrated that SNORA14A upregulation stimulated apoptotic cell death rather than necrotic cell death (Fig. 3F), accompanied by increased cleaved PARP protein expression (Fig. 3G). Moreover, SNORA14A upregulation arrested cells in the G2/M phase (Fig. 3H). Subsequently, we transfected an ASO targeting SNORA14A or a negative control into HB cells and verified the interference efficiency by qRT‒PCR (Fig. S2A). SNORA14A depletion markedly stimulated HB cell proliferation (Fig. S2C–E), reduced cell apoptosis, and promoted G2/M phase transition (Fig. S2F–G). Neither SNORA14A overexpression nor SNORA14A depletion had effects on POR mRNA levels, implying that SNORA14A acts independently of its host gene (Fig. 3B and Fig. S2B). Tumour xenograft assays were conducted to evaluate cell growth in vivo, and the volume and weight of tumours from the SNORA14A-overexpressing group were markedly lower than those of tumours from the control group (Fig. 3I–K). IHC assays confirmed that the percentage of Ki67-positive nuclei was significantly lower in the SNORA14A-overexpressing group than in the control group (Fig. 3L). Collectively, these data suggest that SNORA14A plays a tumour-suppressive role in HB.

### Tumour-suppressive SNORA14A functions by promoting 18 S rRNA maturation in a canonical manner

**Fig. 4:**
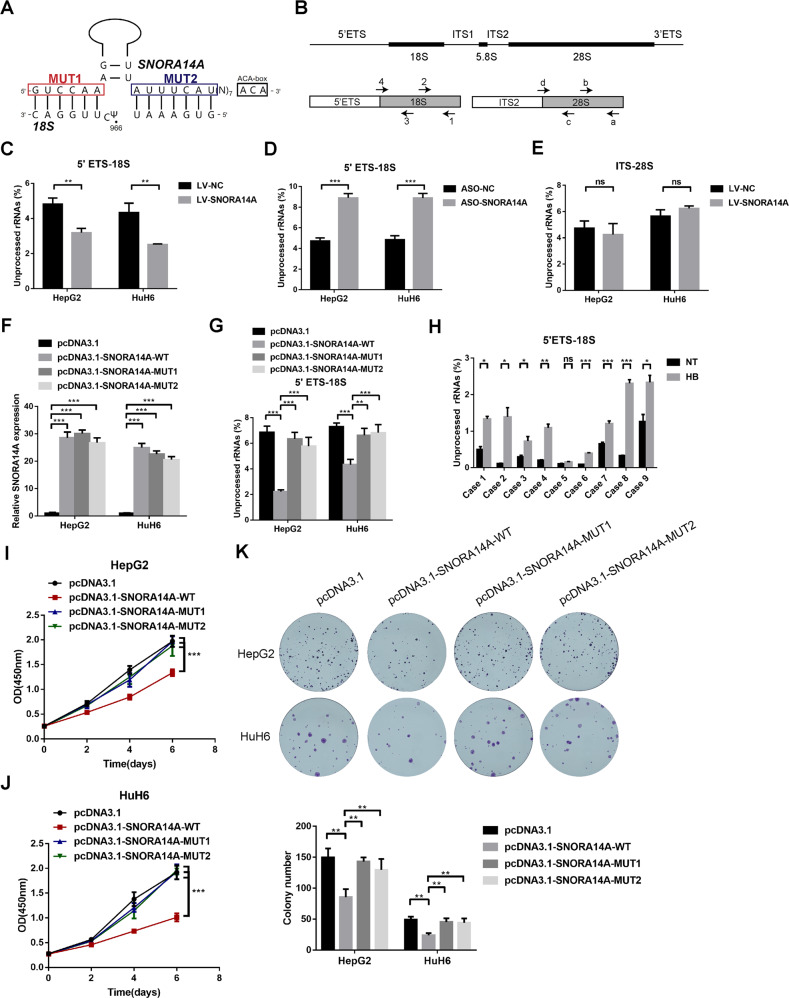
SNORA14A interacts with 18 S rRNA to promote its maturation and inhibit HB cell proliferation. **A** The interaction site between SNORA14A and 18 S rRNA and construction of SNORA14A-mutant vectors. **B** Top: schematic diagram of the eukaryotic rRNA precursor. Bottom: specific primers for total 18 S rRNA (primer pair 1/2), 18 S rRNA precursor (primer pair 3/4), total 28 S rRNA (primer pair a/b) and 28 S rRNA precursor (primer pair c/d). **C**, **D** The percentage of unprocessed 18 S rRNA in HB/LV-NC and HB/LV-SNORA14A cells (**C**) or cells transfected with ASO-NC or ASO-SNORA14A (**D**) was measured by qRT‒PCR. **E** The percentage of unprocessed 28 S rRNA in HB/LV-NC and HB/LV-SNORA14A cells was measured by qRT‒PCR. **F**, **G** Relative expression levels of SNORA14A (**F**) and unprocessed 18 S rRNA (**G**) in treated HB cells were measured by qRT‒PCR. **H** The percentage of unprocessed 18 S rRNA in 9 pairs of HB and NT tissues was measured by qRT‒PCR. **I**–**K** The proliferative activity of treated HB cells was detected by CCK-8 (**I**, **J**) and colony formation (**K**) assays.

SNORA14A canonically guides 18 S rRNA U966 pseudouridylation by directly binding to 18 S rRNA [29]. According to the SnoRNA Atlas Database, the base-pairing interaction between the flanking sequences of 18S-U966 and two antisense elements of SNORA14A forms the structural basis for snoRNA-guided pseudouridylation (Fig. 4A). Considering that pseudouridylation is critical for rRNA maturation [30], we designed specific primers to investigate the role of SNORA14A in rRNA maturation (Fig. 4B) and found that the levels of unprocessed 18 S rRNA were obviously decreased in SNORA14A-overexpressing cells (Fig. 4C) but apparently increased in SNORA14A-depleted cells compared to control cells (Fig. 4D). Unsurprisingly, SNORA14A overexpression had no influence on 28 S rRNA maturation, as 28 S rRNA does not interact with SNORA14A (Fig. 4E). Then, two antisense elements of SNORA14A were separately mutated into corresponding complementary sequences to abrogate its interaction with 18 S rRNA (Fig. 4A). Unprocessed 18 S rRNA levels in cells overexpressing mutant SNORA14A were significantly higher than those in cells overexpressing wild-type SNORA14A (Fig. 4F, G), suggesting that this interaction was indispensable in SNORA14A-mediated 18 S rRNA maturation. Unprocessed 18 S rRNA levels in eight HB tissues were significantly higher than those in paired NT tissues (Fig. 4H), implying that SNORA14A downregulation in HB tissues arrested 18 S rRNA maturation. Interestingly, the proliferative activity of cells overexpressing mutant SNORA14A was also greater than that of cells overexpressing wild-type SNORA14A (Fig. 4I–K). In short, tumour-suppressive SNORA14A functions by promoting 18 S rRNA maturation.

### SNORA14A increases SDHB protein levels by promoting 18 S rRNA maturation

**Fig. 5:**
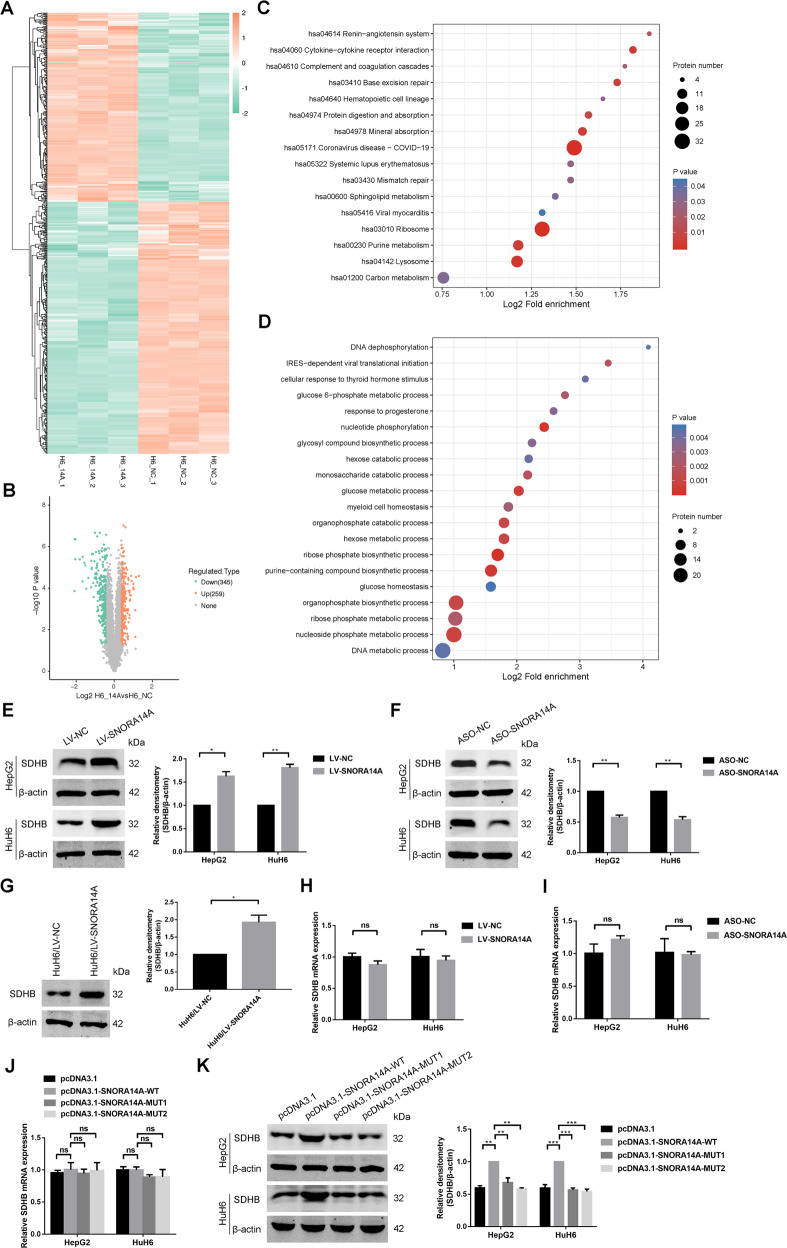
SNORA14A enhances SDHB protein expression by promoting 18 S rRNA maturation. **A** Heatmap clustering of TMT-labelled quantitative proteomics conducted in HuH6/LV-NC and HuH6/LV-SNORA14A cells. **B**, **C** Volcano plot (**B**) and KEGG pathway enrichment analysis (**C**) of significantly differentially expressed proteins. **D** GO enrichment analysis of upregulated proteins. **E**–**G** SDHB protein levels in HB/LV-NC and HB/LV-SNORA14A cells (**E**), cells transfected with ASO-NC or ASO-SNORA14A (**F**) or tumour xenografts (**G**) were measured by Western blotting assays. Relative densitometry was performed with ImageJ. **H**–**J** Relative mRNA levels of SDHB in stable HB cells (**H**) or cells with the indicated transfection condition (**I**, **J**) were measured by qRT‒PCR assays. **K** SDHB protein levels in cells with the indicated transfection condition were measured by Western blotting assays. Relative densitometry was performed with ImageJ.

As the key component of the eukaryotic 40 S ribosomal subunit, 18 S rRNA plays an important role in stabilizing ribosome structure and protein synthesis [31]. To investigate whether SNORA14A might affect specific protein expression by regulating 18 S rRNA maturation, TMT-labelled quantitative proteomics was conducted in HuH6/LV-NC and HuH6/LV-SNORA14A cells, and we identified 604 significantly differentially expressed proteins, including 259 upregulated proteins and 345 downregulated proteins (Fig. 5A, B and Table S2). Functional enrichment analyses showed that ribosomes were enriched in the top 16 KEGG pathways (Fig. 5C). COG/KOG function classification illustrated that 17 upregulated proteins were involved in translation, ribosomal structure, and biogenesis (Fig. S3A). GO enrichment analysis of upregulated proteins confirmed that IRES-dependent viral translation initiation was enriched (Fig. 5D). Strikingly, GO secondary annotation classification showed that 58.7% of upregulated proteins participated in metabolic processes (Fig. S3B). Carbon metabolism, referred to as the carbon utilization pathways of the TCA cycle, glycolysis and pentose phosphate pathway (PPP), was enriched in the top 16 KEGG pathways (Fig. 5C). Then, we focused on the key enzyme of the TCA cycle, SDHB, which ranked ahead in 16 carbon metabolism proteins in terms of fold change (1.507). Western blotting assays validated that SNORA14A overexpression in HB cells elevated SDHB protein levels, while SNORA14A depletion decreased SDHB protein levels (Fig. 5E, F). Accordingly, SDHB protein expression was also increased in SNORA14A-overexpressing tumour xenografts compared to control xenografts (Fig. 5G). However, overexpressing wild-type/mutant SNORA14A or silencing SNORA14A had no effect on SDHB mRNA levels (Fig. 5H–J), suggesting that SNORA14A might affect SDHB expression at the translational or posttranslational level. Furthermore, SDHB protein levels in cells overexpressing mutant SNORA14A were markedly lower than those in cells overexpressing wild-type SNORA14A (Fig. 5K), indicating that 18 S rRNA maturation was involved in SNORA14A-mediated upregulation of SDHB protein. Additionally, quantitative proteomics showed that the level of the GDP-forming β subunit of succinate-CoA ligase (SUCLG2) was slightly decreased in HuH6/LV-SNORA14A cells versus HuH6/LV-NC cells (fold change = 0.705). However, Western blotting assays confirmed that SNORA14A overexpression in HB cells had no apparent effect on SUCLG2 protein levels (Fig. S3C), suggesting that SUCLG2-mediated conversion of succinyl-CoA to succinate is unlikely to be perturbed upon SNORA14A overexpression.

### SDHB suppresses cell proliferation, triggers cell apoptosis and induces G2/M phase arrest

**Fig. 6:**
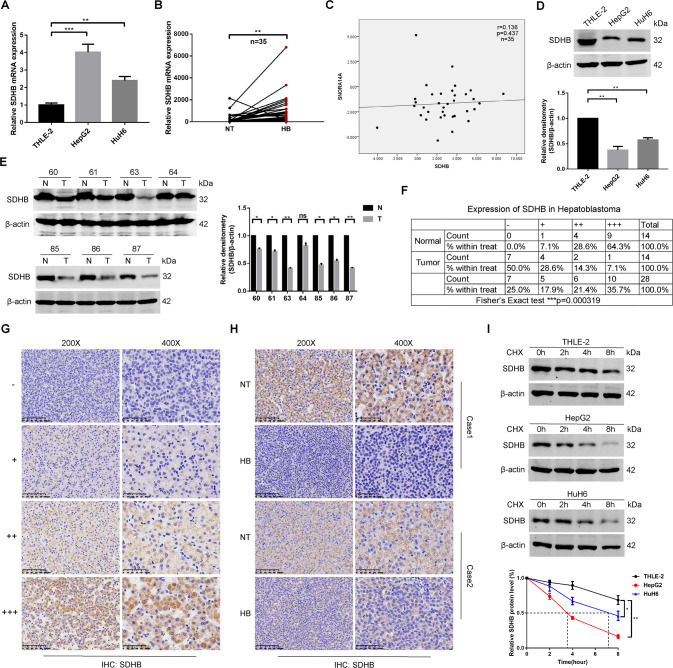
Significant downregulation of SDHB protein levels in HB tissues and cells. **A**, **B** Relative mRNA levels of SDHB in cells (**A**) or 35 pairs of HB and NT tissues (**B**) were measured by qRT‒PCR. **C** Spearman rank correlation analysis of SNORA14A and SDHB expression levels. **D**, **E** SDHB protein levels in cells (**D**) or 7 pairs of HB and NT tissues (**E**) were measured by Western blotting assays. Relative densitometry was performed with ImageJ. **F** Fisher’s exact test was used to analyse SDHB protein levels in 14 pairs of HB and NT tissues. **G**, **H** Representative IHC images of SDHB staining in 14 pairs of HB and NT tissues. **I** SDHB protein levels in THLE-2, HepG2 and HuH6 cells were measured using Western blotting assays after treatment with 100 μg/ml cycloheximide for the indicated times.

qRT‒PCR assays validated the clear increase in SDHB mRNA levels in HB cells and tissues compared to THLE-2 cells and NT tissues (Fig. 6A, B). Consistent with our observation that SNORA14A did not regulate SDHB mRNA expression, Spearman’s rank correlation analysis showed no association between the RNA levels of SNORA14A and SDHB (*r* = 0.136, *P* = 0.437) (Fig. 6C). Moreover, the SDHB protein levels in HB cells and six out of seven HB tissues were clearly lower than those in THLE-2 cells and matched NT tissues (Fig. 6D, E). IHC assays of 14 paired HB and NT tissues showed that the percentages of HB tissues with moderate (++) and strong (+++) cytoplasmic staining intensities of SDHB were lower than those of NT tissues (Fig. 6F–H). Furthermore, protein stability assays confirmed that the half-life of SDHB protein in HB cells was markedly shorter than that in THLE-2 cells (Fig. 6I), suggesting that weaker SDHB protein stability in HB cells might be one reason why mRNA levels were increased but protein levels were discordantly decreased.

**Fig. 7:**
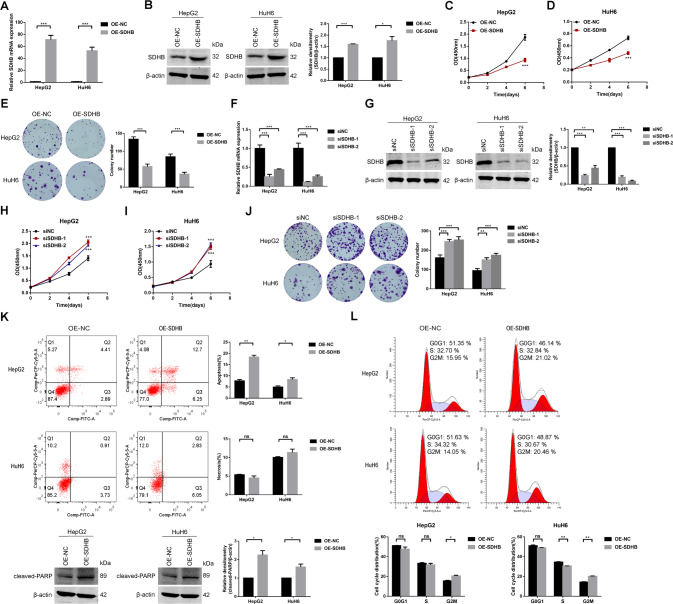
SDHB overexpression inhibits HB cell proliferation, promotes cell apoptosis and arrests cells in the G2/M phase. **A**, **B** The mRNA (**A**) and protein (**B**) levels of SDHB in HB cells transfected with OE-NC or OE-SDHB plasmids were measured by qRT‒PCR and Western blotting assays. Relative densitometry was performed with ImageJ. **C**–**E** The proliferative activity of treated HB cells was detected by CCK-8 (**C**, **D**) and colony formation (**E**) assays. **F**, **G** The mRNA (**F**) and protein (**G**) levels of SDHB in HB cells transfected with siNC or siSDHB-1/-2 were measured by qRT‒PCR and Western blotting assays. Relative densitometry was performed with ImageJ. **H**–**J** The proliferative activity of treated HB cells was detected by CCK-8 (**H**, **I**) and colony formation (**J**) assays. **K** The apoptosis/necrosis and cleaved PARP protein levels of treated HB cells were detected by flow cytometry and Western blotting assays, respectively. Relative densitometry was performed with ImageJ. **L** The cell cycle of treated HB cells was detected by flow cytometry assays.

To explore the biological function of SDHB in HB, we transfected SDHB overexpression or control plasmids into HB cells and verified the overexpression efficiency by qRT‒PCR and Western blotting assays (Fig. 7A, B). Cell proliferation was markedly inhibited after SDHB overexpression (Fig. 7C–E). To clarify whether a mild increase in SDHB expression would still trigger antiproliferative effects, we performed dose-dependent overexpression assays and found that the proliferative activity of cells with different levels of SDHB overexpression was markedly weaker than that of control cells (Fig. S4A–D). Additionally, HB cells were transfected with siRNAs targeting SDHB or a negative control, and the interference efficiency was confirmed (Fig. 7F, G). We found that silencing SDHB stimulated HB cell proliferation (Fig. 7H–J). Moreover, overexpressing SDHB promoted apoptotic cell death but not necrotic cell death, along with increased cleaved PARP protein levels (Fig. 7K) and G2/M phase arrest (Fig. 7L). Collectively, these results indicate that SDHB exerts antiproliferative and proapoptotic effects and induces G2/M phase arrest, consistent with the effects of SNORA14A.

### SNORA14A exerts tumour-suppressive effects in HB by regulating SDHB-mediated succinate metabolism

**Fig. 8:**
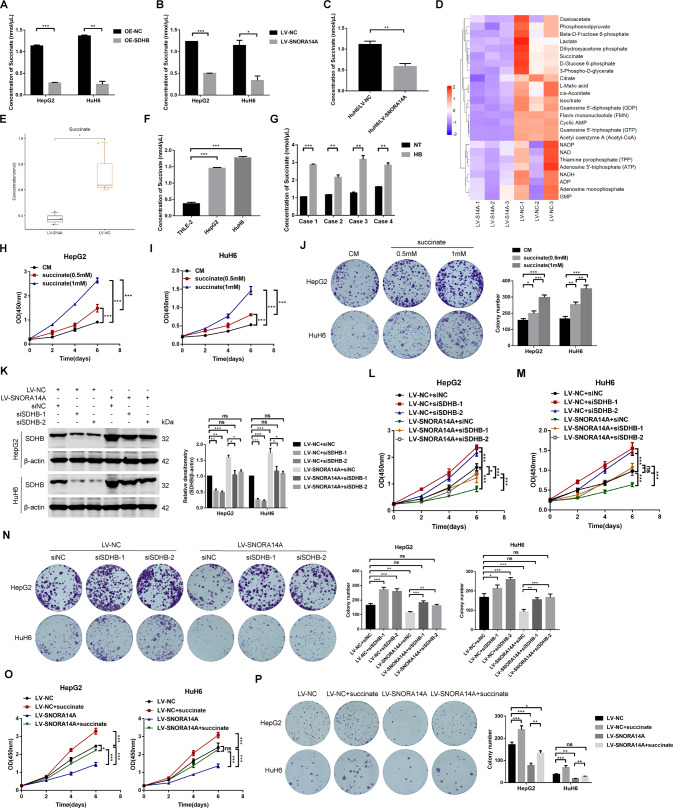
Tumour-suppressive SNORA14A functions by increasing SDHB protein levels and inhibiting accumulation of the oncometabolite succinate in HB cells. **A**–**C** The succinate concentration in HB cells transfected with OE-NC or OE-SDHB plasmids (**A**), stable HB cells (**B**) or tumour xenografts (**C**) was measured by colorimetric assays. **D** Heatmap clustering of targeted metabolomics conducted in stable HuH6 cells. **E** The succinate concentration in stable HuH6 cells was measured by targeted metabolomics. **F**, **G** Succinate concentrations in cells (**F**) or 4 paired HB and NT tissues (**G**) were measured by colorimetric assays. **H**–**J** The proliferative activity of HB cells treated with the indicated concentration of succinate was detected by CCK-8 (**H**, **I**) and colony formation (**J**) assays. **K** SDHB protein levels in stable HB cells transfected with siNC or siSDHB-1/-2 were measured by Western blotting assays. Relative densitometry was performed with ImageJ. **L**–**N** The proliferative activity of stable HB cells transfected with siNC or siSDHB-1/-2 was detected by CCK-8 (**L**, **M**) and colony formation (**N**) assays. **O**, **P** The proliferative activity of stable HB cells supplemented with succinate was detected by CCK-8 (**O**) and colony formation (**P**) assays.

SDHB facilitates the oxidization of succinate into fumarate in the TCA cycle [15]. Colorimetric assays showed that succinate levels in HB cells overexpressing SDHB were significantly lower than those in control cells (Fig. 8A). We speculated that SNORA14A might affect succinate metabolism via SDHB. The results showed that SNORA14A overexpression markedly decreased cellular succinate levels (Fig. 8B). Consistently, succinate reduction was detected in SNORA14A-overexpressing tumour xenografts compared to control xenografts (Fig. 8C). Given that electron transport is directly linked to SDHB [15], we also detected the effects of overexpression of SNORA14A and SDHB on cellular ROS levels and found that ROS levels were decreased in HB cells with overexpression of these factors (Fig. S5A). To further study the role of SNORA14A in energy metabolism, targeted metabolomics was performed on HuH6 LV/NC and HuH6 LV/SNORA14A cells, and the results revealed significant changes in 13 energy metabolites (Fig. 8D and Table S3), including metabolites of the TCA cycle, oxidative phosphorylation, glycolysis, and purine metabolism, revealing the broad regulatory roles of SNORA14A in energy metabolism. Consistently, metabolomics analysis confirmed that SNORA14A overexpression decreased succinate levels in HuH6 cells (Fig. 8E). Moreover, succinate levels were increased in HB cells and tissues compared to THLE-2 cells and paired NT tissues (Fig. 8F, G), indicating that SNORA14A depletion in HB contributes to aberrant succinate accumulation. To examine the biological function of succinate, HB cells were treated with 0, 0.5 or 1 mM succinate and subjected to CCK-8 and colony formation assays. As shown in Fig. 8H–J, succinate induced HB cell proliferation in a concentration-dependent manner.

To further investigate whether SNORA14A influenced HB progression via SDHB and succinate metabolism, HB cells stably overexpressing SNORA14A were transfected with SDHB siRNAs, and Western blotting assays confirmed that the upregulation of SDHB induced by SNORA14A overexpression was blocked by SDHB knockdown (Fig. 8K). The decrease in succinate and ROS levels induced by SNORA14A overexpression could be rescued by SDHB depletion (Fig. S5B, C). Functionally, both silencing SDHB and replenishing succinate restored the decrease in cell proliferative activity (Fig. 8L–P), the increase in cell apoptosis (Fig. S6A and Fig. S7A) and G2/M phase arrest (Fig. S6B and Fig. S7B) induced by SNORA14A overexpression. Thus, tumour-suppressive SNORA14A increases SDHB protein levels to prevent oncometabolite succinate accumulation in HB cells.

### SNORA14A and SDHB are potential diagnostic and prognostic biomarkers for HB

To evaluate the diagnostic value of SNORA14A in HB, we performed ROC curve analysis and found that SNORA14A was able to distinguish 35 HB tissues from 35 NT tissues (AUC = 0.7135; *P* = 0.0021) (Fig. S8). Next, Fisher’s exact test was applied to analyse the association between SNORA14A or SDHB expression and the clinicopathological characteristics of HB patients. The clinicopathological information of 35 HB patients is shown in Table S4. Thirty-five pairs of tissue specimens were divided into high or low SNORA14A expression groups according to the median ΔΔCT value of SNORA14A from qRT‒PCR assays. For 14 pairs of tissue specimens detected by IHC assays, specimens with weak (− or + ) and strong (++ or +++) staining intensity were classified into low and high SDHB expression groups, respectively. A strong correlation was observed between SNORA14A expression level and prognostic factors, including PRETEXT stage (*P* = 0.010) and metastasis (*P* = 0.001) (Table S5). The SDHB protein level was significantly correlated with PRETEXT stage (*P* = 0.011), metastasis (*P* = 0.027), and SNORA14A expression (*P* = 0.011) (Table S6). Hence, SNORA14A and SDHB are promising diagnostic and prognostic biomarkers for HB patients.

## Discussion

Researchers have recently discovered that, beyond genetic mutations, dysregulation of ncRNAs is central to the occurrence and progression of HB [32–34]. Aberrant expression of snoRNAs drives the malignant phenotype of multiple cancers [27, 28]. However, the expression profile and function of snoRNAs in HB have never been investigated. This study first identified a total of 72 differentially expressed snoRNAs in four HB tissues versus NT tissues by snoRNA sequencing and confirmed that SNORA14A downregulation in HB tissues was closely correlated with advanced PRETEXT stage and metastasis. The relationship between SNORA14A and cancer remains largely unknown. Here, we demonstrated that SNORA14A significantly suppresses HB cell proliferation and induces cell apoptosis and G2/M phase arrest in a manner independent of its parental gene POR.

Canonical box H/ACA snoRNAs guide rRNA precursor pseudouridylation and subsequent maturation by directly binding to rRNA [35]. Here, we confirmed that SNORA14A promotes 18 S rRNA maturation via base-pairing interaction with the flanking sequences of 18S-U966, a reported pseudouridylation site guided by SNORA14A [29]; consequently, SNORA14A-deficient HB cells and tissues display delayed 18 S rRNA maturation. Moreover, SNORA14A suppresses HB progression by interacting with 18 S rRNA and promoting its maturation. Current studies have indicated that 18 S rRNA pseudouridylation preferentially regulates IRES-dependent translation initiation but not cap-dependent translation initiation and therefore has no influence on overall protein synthesis [36, 37]. 18S-U966 is located in a reported active region for the translation of IRES-containing mRNAs [38]. Our results indicate that SNORA14A upregulates certain proteins involved in ribosomal structure, biogenesis and IRES-dependent translation initiation but does not apparently increase overall protein levels. Indeed, the overwhelming majority of 18 S rRNAs still matured and worked in HB cells. Consequently, overexpressing SNORA14A in HB cells promoted the maturation of the relatively few unprocessed 18 S rRNAs but did not enhance ribosome function to a level sufficient to induce an apparent increase in overall protein levels. Numerous studies have demonstrated that loss of individual or limited numbers of snoRNAs is unable to perturb ribosome performance to yield overall translation defects because the remaining snoRNAs still sustain ribosome function [35, 39], which is consistent with our conclusions.

Accumulating studies have revealed the noncanonical regulatory roles of box C/D snoRNAs in cellular metabolism [25, 26]. However, the function of box H/ACA snoRNAs in metabolic processes is largely unknown. Here, proteomics analysis suggested that SNORA14A played expansive roles in various metabolic processes, especially in carbon metabolism. SNORA14A positively regulated SDHB expression at the protein level but not the mRNA level by promoting 18 S rRNA maturation. 18 S rRNA facilitates the interaction of ribosomes with specific mRNAs and translation factors [31]. Here, proteomics analysis showed that several eukaryotic translation initiation factors (EIF4G1, EIF2D, EIF3M, EIF5A) were markedly upregulated by SNORA14A. The interaction between eIF4G1 protein and SDHB mRNA has been revealed by an iCLIP-seq study [40]. In the future, we will investigate whether SNORA14A can regulate SDHB mRNA translation initiation.

Dysregulation of cellular energetics is a hallmark of cancer [41]. Reprogramming of glucose, glutamine and lipid metabolism provides growth advantages to HB cells [11–13]. Here, we found that downregulation of SDHB protein in HB tissues was markedly correlated with advanced PRETEXT stage, metastasis and low SNORA14A expression. Comparable to SNORA14A, SDHB also exerted antiproliferative and proapoptotic effects and induced G2/M phase arrest. It is reported that SDHB depletion in cancer cells causes succinate accumulation and activation of several oncogenic signalling pathways [17, 19, 42]. Loss of SDHB induces ROS production and subsequently stabilizes HIF-α, promoting osteosarcoma and lung cancer cell growth [43]. Here, we proved that SNORA14A-triggered upregulation of SDHB reduced the levels of two well-known carcinogens, succinate and ROS. SNORA14A-deficient HB cells and tissues displayed abnormal accumulation of succinate, which could aggravate HB malignant phenotypes; however, the exact mechanisms require further investigation. Additionally, SDHB deficiency switches energy metabolism from aerobic respiration to glycolysis, thus accelerating tumour cell proliferation or metastasis [16, 44]. Here, targeted metabolomics revealed a reduction in lactate levels upon SNORA14A overexpression. Despite the upregulation of hexokinase 2, which phosphorylates glucose to glucose 6-phosphate for glycolysis and PPP [45], the increase in SDHB as well as the decrease in the glycolysis-promoting phosphoenolpyruvate carboxykinase 2 (PCK2) and aldehyde dehydrogenase 2 (ALDH2) [46, 47] might coordinately lead to this phenomenon (see Table S2). The potential regulatory roles of SNORA14A in glycolysis will be investigated in future studies.

In summary, we provide the first evidence that SNORA14A expression is significantly decreased in HB tissues and is associated with PRETEXT stage and metastasis. SNORA14A prohibits HB cell proliferation and induces apoptosis and G2/M phase arrest. Mechanistically, SNORA14A promotes 18 S rRNA precursor maturation to elevate SDHB protein expression, thereby preventing aberrant accumulation of the oncometabolite succinate in HB cells. The critical role of the SNORA14A/18 S rRNA/SDHB pathway makes it a promising prognostic biomarker and attractive therapeutic target for HB patients.

## Materials and Methods

### HB patients and clinical specimens

Paired HB tissues and adjacent NT liver tissues from 35 HB patients undergoing hepatectomy without preoperative chemotherapy or radiotherapy were obtained from Shanghai Children’s Medical Centre (Shanghai, China). Two pathologists confirmed that NT tissues, which were resected 3 cm away from the tumour margin, excluded tumour cells. Clinicopathological information was available for patients providing each specimen. This study was approved by the ethics committee of Shanghai Children’s Medical Centre. Written informed consent was obtained from every participant.

### Cell culture and treatment

A human normal hepatocyte line (THLE-2) was purchased from Ningbo Mingzhou Biotechnology Co., Ltd. (Ningbo, China). THLE-2 cells were cultured in BEBM medium (CC3170, Lonza/Clonetics Corporation, Walkersville, MD, USA) supplemented with 5 ng/mL EGF (SRP3027, Sigma‒Aldrich), 70 ng/mL phosphorylethanolamine (HY-N5034, MedChemExpress, Monmouth Junction, NJ, USA), 10% foetal bovine serum (FBS, 10099141, Gibco Laboratories, Gaithersburg, MD, USA) and 1% penicillin–streptomycin solution (C100C5, NCM Biotech, Suzhou, China) in a 5% CO_2_ incubator at 37 °C. Human HB cell lines (HepG2 and HuH6) were obtained from the Cell Bank of the Type Culture Collection of the Chinese Academy of Sciences (Shanghai, China). HepG2 and HuH6 cells were separately cultured in MEM (SH30265.01, HyClone, Logan, UT, USA) or DMEM (C11995500BT, Gibco Laboratories) supplemented with 10% FBS and 1% penicillin–streptomycin solution in a 5% CO_2_ incubator at 37 °C. For RNA decay assays, cells were treated with 5 μg/ml actinomycin D (HY-17559, MedChemExpress) for 0, 30, 60, and 90 min. For protein stability assays, cells were treated with 100 μg/ml cycloheximide (HY-12320, MedChemExpress) for 0, 2, 4, and 8 h. For some assays, cells were treated with culture medium (CM) or succinate (S9512, Sigma‒Aldrich) for the desired time.

### Cell proliferation assay

For the CCK-8 assay, 1000 cells were seeded in each well of a 96-well plate. On the indicated days, the medium was changed to 100 μL of fresh medium containing 10 μL of CCK-8 reagent (C0043, Beyotime Biotechnology, Shanghai, China), and the cells were incubated for 2 h at 37 °C. A Synergy2 multimode microplate reader (BioTek Instruments, Winooski, VT, USA) was used to measure the absorbance at 450 nm.

For the colony formation assay, 1000 cells were seeded in each well of a 12-well plate. After culturing for 7–10 days, the cells were washed with PBS, fixed with 4% paraformaldehyde solution, and then stained with 0.1% crystal violet dye. Finally, cell colonies were photographed and automatically calculated by ImageJ (U.S. National Institutes of Health, Bethesda, MD, USA).

### Flow cytometry

For cell apoptosis analysis of HB/LV-NC or HB/LV-SNORA14A cells, an Annexin V–APC/7-AAD apoptosis kit (AP105, MultiSciences Biotech Co., Ltd., Hangzhou, China) was used. Approximately 2 × 10^5^ cells were harvested, washed with PBS, and resuspended in 500 μL of 1× binding buffer. Cells were then stained with 5 μL of Annexin V–APC and 10 μL of 7-AAD for 5 min in the dark at room temperature. For cells subjected to other indicated treatments, an Annexin V–FITC apoptosis detection kit (C1062L, Beyotime Biotechnology) was used. Cells were resuspended in 195 μL of Annexin V–FITC binding buffer and then stained with 5 μL of Annexin V–FITC and 10 μL of propidium iodide for 20 min in the dark at room temperature. For cell cycle analysis, a cell cycle and apoptosis analysis kit (C1052, Beyotime Biotechnology) was used. Cells were synchronized in medium without FBS for 12 h, followed by recovery in fresh medium for another 12 h. Then, the cells were harvested, washed with PBS, and fixed with 1 mL of 70% ethanol overnight at 4 °C. The next day, the cells were washed with PBS and stained with 400 μL of staining buffer, 20 μL of propidium iodide, and 8 μL of ribonuclease A for 30 min in the dark at 37 °C. FACS flow cytometry (BD Biosciences, San Jose, CA, USA) was used to detect the percentage of apoptotic cells or cell cycle distribution.

### TMT-labelled quantitative proteomics

TMT-labelled quantitative proteomics was conducted as previously described [48]. For detailed information, see the supplemental materials and methods.

### Targeted metabolomics analysis

Targeted metabolomics analysis was conducted as previously reported [49]. For detailed information, see the supplemental materials and methods.

### Statistical analysis

SPSS Statistics 24.0 (IBM Corporation, Armonk, NY, USA) and GraphPad Prism 7.0 (GraphPad Software, San Diego, CA, USA) were used for data analysis. Data from three independent assays or six mice per group in the animal assays were used to determine the mean ± STD. The statistical significance in two-group comparisons was analysed using an independent or paired-samples *t* test, while comparisons among more than three groups were calculated by one-way ANOVA (Bonferroni’s test or Dunnett’s test). Two-way ANOVA (Bonferroni’s test) was used to analyse the tumour growth curve. Fisher’s exact test was applied to analyse the correlation of SNORA14A or SDHB expression and clinicopathological characteristics of HB patients. ROC curves were used to evaluate the diagnostic value of SNORA14A in HB. Statistical significance is represented as *P < 0.05, **P < 0.01, or ***P < 0.001.

For further detailed materials and methods, see the supplementary materials and methods.

## Supplementary information

Supplementary materials and methods

Supplementary figure legends

Figure S1

Figure S2

Figure S3

Figure S4

Figure S5

Figure S6

Figure S7

Figure S8

Table S1

Table S2

Table S3

Table S4

Table S5

Table S6

Original Western blotting images

## Data Availability

The data generated in this study are available within the article and its supplementary data files. The raw sequence data reported in this paper have been deposited in the Genome Sequence Archive (Genomics, Proteomics & Bioinformatics 2021) in the National Genomics Data Centre (Nucleic Acids Res 2021), China National Centre for Bioinformation/Beijing Institute of Genomics, Chinese Academy of Sciences (GSA: HRA003219) and are publicly accessible at https://ngdc.cncb.ac.cn/gsa.
