# Supplementary material for: SNORA14A inhibits hepatoblastoma cell proliferation by regulating SDHB-mediated succinate metabolism
Source: Cell Death Discov. 2023 Jan 30;9:36. doi: 10.1038/s41420-023-01325-0 (PMC9886955; doi:10.1038/s41420-023-01325-0)
Supplement: Supplementary file 1 — Supplementary materials and methods [file 41420_2023_1325_MOESM1_ESM.docx]

**Supplementary Materials and Methods**

**SnoRNA sequencing**

SnoRNA sequencing of 4 paired HB and adjacent NT tissues was conducted by Aksomics Co., Ltd. (Shanghai, China). Total RNA samples were quantified using a NanoDrop ND-1000 instrument and then sequentially ligated to 3' and 5' small RNA adapters. cDNA was synthesized and amplified using Illumina’s proprietary RT primers and amplification primers. Subsequently, ~170-370 bp PCR amplified fragments were extracted and purified from the PAGE gel. The prepared snoRNA-seq libraries were quantified using an Agilent 2100 Bioanalyzer and then sequenced using an Illumina NovaSeq 6000 (Illumina, Inc., San Diego, CA, USA). Sequencing quality was examined by FastQC software [http://www.bioinformatics.babraham.ac.uk/projects/fastqc/], and trimmed reads (pass Illumina quality filter, trimmed 3'-adaptor bases by cutadapt [1]) were aligned to Ensemble (a manually curated resource of snoRNAs) using NovoAlign software (v2.07.11) [http://www.novocraft.com/main/index.php]. The maximum mismatch ≤ 0 reads to the Ensemble were kept. The expression profiling and differential expression of snoRNAs were calculated based on normalized TPM[2]. SnoRNAs with fold changes ≥ 1.5 or fold changes ≤ 0.67 and P value ≤ 0.05 were selected as differentially expressed snoRNAs. Hierarchical clustering, scatter plots and classification analysis were performed with differentially expressed snoRNAs in the R or Perl environment for statistical computing and graphics. Kyoto Encyclopedia of Genes and Genomes (KEGG) and DAVID databases were applied for signalling pathway and Gene Ontology (GO) analyses, respectively.

**Oligonucleotide or plasmid transfection and lentiviral transduction**

The antisense oligonucleotide (ASO) against SNORA14A and small interfering RNAs (siRNAs) against SDHB were purchased from RiboBio Corporation (Guangzhou, China) and GenePharma Corporation (Shanghai, China), respectively. The sequences used were as follows: ASO-NC (sequences offered by RiboBio Corporation), ASO-SNORA14A (target sequence: 5'-CCCTGTAAATGCTTCCAAGA-3'), siNC (sense: 5'-UUCUCCGAACGUGUCACGUTT-3', antisense: 5'-ACGUGACACGUUCGGAGAATT-3'), siSDHB-1 (sense: 5'-CAAGAAAUUUGCCAUCUAUTT-3', antisense: 5'-AUAGAUGGCAAAUUUCUUGTT-3'), and siSDHB-2 (sense: 5'-CCACACAUGUAUGUGAUAATT-3', antisense: 5'-UUAUCACAUACAUGUGUGGTT-3'). The wild-type SNORA14A and mutant SNORA14A expression vectors and SDHB overexpression vector were purchased from Tsingke Biotechnology Corporation (Beijing, China). For construction of the pcDNA3.1-SNORA14A-WT vector, the SNORA14A coding sequence was cloned into the pcDNA3.1(-) vector. For construction of the pcDNA3.1-SNORA14A-MUT1 vector, antisense element 1 (GUCCAA) was mutated into “CAGGUU”. For construction of the pcDNA3.1-SNORA14A-MUT2 vector, antisense element 2 (AUUUCAU) was mutated to “UAAAGUA”. For construction of HB cells stably overexpressing SNORA14A, the SNORA14A coding sequence, which was constructed into the pGMLV-CMV-MCS-EF1-ZsGreen1-T2A-Puro vector, was purchased from Ke Lei Biotechnology Corporation (Shanghai, China). HB cells were transfected with ASOs, siRNAs, or plasmids using the riboFECT^TM^ CP transfection kit (C10511-05, RiboBio) or Lipofectamine™ 2000 transfection reagent (11668019, Invitrogen, Carlsbad, CA, USA) in accordance with the manufacturer's procedures.

**RNA extraction and real-time quantitative polymerase chain reaction (qRT‒PCR)**

TRIzol reagent (15596026, Invitrogen) was used to extract total RNA from tissues or whole-cell lysates. The PrimeScript™ RT reagent kit with gDNA eraser (RR047A, TaKaRa Bio, Kusatsu, Japan) and TB Green™ Premix Ex Taq™ II (RR820A, TaKaRa Bio) were used to detect the abundance of SNORA14A, mRNAs, and unprocessed rRNAs. The 2^−ΔΔCT^ method was used for relative quantitation, and the ΔΔCT method was adopted for Spearman rank correlation analysis [3]. U6 snRNA was used as an internal control to standardize SNORA14A expression, and 18S rRNA was used as an internal control to standardize mRNA expression. The percentage of unprocessed rRNAs (5'ETS-18S and ITS2-28S) was measured by calculating the averages of primer pairs for unprocessed 18S/28S rRNA over total 18S/28S rRNA [4]. The primer pairs used were as follows: SNORA14A (forward: 5'-TTGGTGGCTTCCCTGTAAATG-3', reverse: 5'-GAAATAAGACTGAGCCACAGGAG-3'); POR (forward: 5'-GCGGTGGCCGAAGAAGTAT-3', reverse: 5'-TGACAGAGGAGGTCAATGTCTG-3'); SDHB (forward: 5'-CACTCTAGCTTGCACCCGAA-3', reverse: 5'-CGTCCAGTTTCTCACGCTCT-3'); unprocessed 18S rRNA (forward: 5'-CTCGCCGCGCTCTACCTTACCTACCTGG-3', reverse: 5'-GCGCCCGTCGGCATGTATTAGCTC-3'); total 18S rRNA (forward: 5'-GGCCCTGTAATTGGAATGAG-3', reverse: 5'-GCGGGACACTCAGCTAAGAGC-3'); unprocessed 28S rRNA (forward: 5'-CCCGTCCCCCTCCGAGACGCGACC-3', reverse: 5'-CGCTGGGCTCTTCCCTGTTCACTCG-3'); total 28S rRNA (forward: 5'-CCAAGTCCTTCTGATCGAGGCCC-3', reverse: 5'-CTTACGGTACTTGTTGACTATCGGTCTCG-3'); 18S rRNA (forward: 5'-CAGCCACCCGAGATTGAGCA-3', reverse: 5'-TAGTAGCGACGGGCGGTGTG-3'); and U6 snRNA (RT: 5'-AAAATATGGAACGCTTCACGAATTTG-3', forward: 5'-CTCGCTTCGGCAGCACA-3', reverse: 5'-AACGCTTCACGAATTTGCGT-3').

**Western blotting assay**Western blotting assays were conducted according to a previously reported protocol [5]. The primary anti-SDHB antibody (178423, Abcam, Cambridge, UK), primary cleaved-PARP antibody (5625T, Cell Signaling Technology, Danvers, MA, USA), primary anti-SUCLG2 antibody (187996, Abcam), primary anti-β-actin antibody (8226, Abcam), primary anti-GAPDH antibody (8245, Abcam) or IRDye® 800CW goat anti-mouse/rabbit IgG secondary antibodies (926-32210, 926-32211, LI-COR Biosciences, Lincoln, NE, USA) were used. The bands on the membranes were visualized with an Odyssey infrared imaging system (LI-COR Biosciences). ImageJ was utilized for quantification of the integrated optical density of the bands.

**Immunohistochemistry (IHC)**

IHC assays were performed on the tissue microarray of 14 pairs of HB tissues and matched NT tissues. The tissue microarray was subjected to deparaffinization, hydration, antigen retrieval, and blockade of endogenous peroxidase and nonspecific staining, followed by incubation with primary anti-SDHB antibody (178423, Abcam) overnight at 4 °C and biotinylated goat anti-rabbit IgG secondary antibody and peroxidase-labelled streptavidin for 15 min at room temperature. Afterwards, the tissue microarray was incubated with diaminobenzidine chromogenic substrate for 10 min at room temperature. Runnerbio Corporation (Shanghai, China) conducted panoramic scanning of the tissue microarray. Two specialized pathologists completely blinded to the clinicopathological information of the tissue specimens performed staining intensity analyses at 200× and 400× magnifications.

**Tumour xenograft assay**

Four-week-old male nude mice (n = 6) were acquired from Shanghai Super-B&K Laboratory Animal Corporation (Shanghai, China) and maintained in the SPF animal house of Shanghai Children’s Medical Center. We subcutaneously injected 1×10^7^ HuH6/LV-NC and HuH6/LV-SNORA14A cells into the left or right flank of each mouse and then measured the length and width of tumours every 4 days. The tumour volume was calculated with the following formula: tumour volume (mm^3^) = 0.5 × length (mm) × width^2^ (mm^2^). After 28 days, the mice were sacrificed, and their tumours were dissected, weighed, and photographed. IHC assays of Ki67 in dissected tumours were conducted by Runnerbio (Shanghai, China). The animal assays were performed according to the Guidelines for the Care and Use of Animals for Scientific Research.

**TMT-labelled quantitative proteomics**

TMT-labelled quantitative proteomics of HuH6/LV-NC and HuH6/LV-SNORA14A cells was conducted by PTM BioLab Corporation (Hangzhou, China). Briefly, the samples were dissolved in lysate buffer consisting of 1% protease inhibitor, 8 M urea, 50 mM NAM, and 3 μM TSA using an ultrasonic processor for 10 min on ice. Then, the supernatant was collected by centrifugation at 12000 × g for 10 min at 4 °C and quantified with a bicinchoninic acid protein assay kit. The protein samples were treated with 5 mM DTT for 30 min at 56 °C, followed by 11 mM iodoacetamide for 15 min in the dark at room temperature. After trypsin digestion overnight, the peptides were desalted with a Strata X C18 SPE column (Phenomenex, Torrance, CA, USA), vacuum-dried, and then reconstituted with 0.5 M TEAB and labelled with the TMT kit. Liquid phases A and B were 0.1% formic acid and 90% acetonitrile containing 0.1% formic acid, respectively. For liquid chromatography (LC)–tandem mass spectrometry (MS) analysis, tryptic peptides were dissolved in liquid phase A, loaded on a reversed-phase analytical column and separated with the EASY-nLC 1200 ultra-performance LC system (Thermo Fisher Scientific). The gradient of liquid phase B was set as 0–40 min at 6%–22%, 40–54 min at 22%–32%, 54–57 min at 32%–80%, or 57–60 min at 80%. The flow rate was 500 nL/min. Afterwards, the peptides were subjected to Q Exactive tandem MS (Thermo Fisher Scientific). The electrospray voltage was 2.1 kV, and an Orbitrap ion trap mass analyser (Thermo Fisher Scientific) was utilized for detection. The primary MS system scanned from 350–1,600 m/z, while the secondary tandem MS system scanned a startup of 100 m/z. A data-dependent procedure was used with 30 s of dynamic exclusion. The automatic gain control was set to 1E5. MS2 data were retrieved with MaxQuant software (Max-Planck-Institute of Biochemistry, Planegg, Germany). For bioinformatics analysis, the UniProt-GOA database was adopted for GO annotation of the proteome. Protein pathways were annotated with the KEGG database. For enrichment analysis of GO or KEGG pathways, the enrichment of differentially expressed proteins against all identified proteins was analysed by two-tailed Fisher’s exact test. The threshold for screening significantly differentially expressed proteins was *P* < 0.05 and a fold change of >1.3 or <0.77.

**Targeted metabolomics analysis**

The targeted metabolomics analysis was conducted by Applied Protein Technology Corporation (Shanghai, China) with the Agilent 1290 Infinity LC system (Agilent Technologies) and 5500 QTRAP MS (AB SCIEX, Framingham, MA, USA). Briefly, mobile phases A and B were ammonium acetate (10 mM) and acetonitrile, respectively. The supernatant extracted from HuH6/LV-NC or HuH6/LV-SNORA14A cells was transferred into a 4 °C automatic sampler with a 45 °C column temperature, 2-μL injection volume, and 300-μL/min flow rate. The gradient of acetonitrile was set as 0–18 min at 90%–40%, 18–18.1 min at 40%–90%, or 18.1–23 min at 90%. The MS system with a negative ion mode was operated as follows: IonSpray voltage floating, −4500 V; source temperature, 450 °C; ion source gas 1, 45 psi; ion source gas 2, 45 psi; and curtain gas, 30 psi. Analysis was performed by electrospray ionization with multireaction monitoring. MultiQuant software (AB SCIEX) was applied to determine the peak chromatographic area and retention time. Finally, standards of energy metabolites were applied for retention time calculation and metabolite identification.

**Colorimetric assay**

Succinate levels in tissues or cells were measured using a succinate assay kit (ab204718, Abcam) according to the manufacturer's procedure. Briefly, freshly diluted succinate standard solutions were prepared and kept on ice. Then, 10 mg of tissues or 1×10^6^ cells were harvested, washed with precooled PBS, resuspended in 100 µL of precooled succinate assay buffer, and rapidly homogenized on ice. Afterwards, the samples were centrifuged at 14,000 rpm for 5 min at 4 °C to remove insoluble material. The supernatant was spun and filtered using a 10-kDa spin column and kept on ice. Subsequently, 50 µL of standard dilutions or samples (adjusted to 50 µL with succinate assay buffer) were added to each well of a 96-well plate, and 50 µL of reaction mix (42 µL of succinate assay buffer, 2 µL of succinate converter, 2 µL of succinate enzyme mix, 2 µL of succinate substrate mix, and 2 µL of succinate developer) was added to each well, followed by incubation at 37 °C for 30 min in the dark. Ultimately, the Synergy2 multimode microplate reader (BioTek Instruments) measured the absorbance at a wavelength of 450 nm. Succinate was measured using the following formula: succinate concentration (nmol/µL) = amount of succinate from standard curve (nmol)/sample volume added to each well (µL) × sample dilution factor.

**Measurement of intracellular ROS levels**

The ROS levels of cells with the indicated transfection condition were detected with a Reactive Oxygen Species Assay Kit (S0033S, Beyotime Biotechnology). Briefly, cells were harvested and incubated with 2',7'-dichlorofluorescein-diacetate (DCFH-DA) diluted in FBS-free medium for 20 min at 37 °C, followed by three washes with FBS-free medium. Finally, the cell suspension was subjected to FACS flow cytometry.

# References

1. Martin, M., Cutadapt removes adapter sequences from high-throughput sequencing reads. Embnet Journal, 2011. **17**(1).

2. Bullard, J.H., E. Purdom, K.D. Hansen, and S. Dudoit, Evaluation of statistical methods for normalization and differential expression in mRNA-Seq experiments. BMC Bioinformatics, 2010. **11**: p. 94.

3. Yuan, J.S., A. Reed, F. Chen, and C.N. Stewart, Jr., Statistical analysis of real-time PCR data. BMC Bioinformatics, 2006. **7**: p. 85.

4. Cao, P., A. Yang, R. Wang, X. Xia, Y. Zhai, Y. Li, et al., Germline Duplication of SNORA18L5 Increases Risk for HBV-related Hepatocellular Carcinoma by Altering Localization of Ribosomal Proteins and Decreasing Levels of p53. Gastroenterology, 2018. **155**(2): p. 542-556.

5. Zhen, N., S. Gu, J. Ma, J. Zhu, M. Yin, M. Xu, et al., CircHMGCS1 Promotes Hepatoblastoma Cell Proliferation by Regulating the IGF Signaling Pathway and Glutaminolysis. Theranostics, 2019. **9**(3): p. 900-919.
