# Supplementary material for: SNORA14A inhibits hepatoblastoma cell proliferation by regulating SDHB-mediated succinate metabolism
Source: Cell Death Discov. 2023 Jan 30;9:36. doi: 10.1038/s41420-023-01325-0 (PMC9886955; doi:10.1038/s41420-023-01325-0)
Supplement: Supplementary file 2 — Supplementary figure legends [file 41420_2023_1325_MOESM2_ESM.docx]

**Supplementary Figure legends**

**Figure S1: HB cells with different levels of SNORA14A overexpression possess apparently weaker proliferative activity than control cells.** (A) Relative expression levels of SNORA14A in HB cells transfected with the indicated amounts of OE-SNORA14A or OE-NC plasmids were measured by qRT‒PCR. (B-D) The proliferative activity of HB cells transfected with the indicated amounts of plasmids was detected by CCK-8 (B-C) and colony formation (D) assays.

**Figure S2: Knockdown of SNORA14A promotes HB cell proliferation, inhibits cell apoptosis and induces G2/M phase transition.** (A-B) Relative expression levels of SNORA14A (A) and POR (B) in HB cells transfected with ASO-NC or ASO-SNORA14A were measured by qRT‒PCR. (C-E) The proliferative activity of HB cells transfected with ASO-NC or ASO-SNORA14A was detected by CCK-8 (C-D) and colony formation (E) assays. (F) Apoptosis and necrosis of HB cells transfected with ASO-NC or ASO-SNORA14A were detected by flow cytometry assays. (G) The cell cycle of HB cells transfected with ASO-NC or ASO-SNORA14A was detected by flow cytometry assays.

**Figure S3:** Functional classification of proteins upregulated by SNORA14A overexpression. (A-B) COG/KOG function classification (A) and GO secondary annotation classification (B) of proteins upregulated by SNORA14A overexpression. (C) SUCLG2 protein levels in HB/LV-NC and HB/LV-SNORA14A cells were measured by Western blotting assays. Relative densitometry was performed with ImageJ.

**Figure S4: HB cells with different levels of SDHB overexpression exhibit apparently weaker proliferative activity than control cells.** (A) Relative expression levels of SDHB in HB cells transfected with the indicated amounts of OE-SDHB or OE-NC plasmids were measured by qRT‒PCR. (B-D) The proliferative activity of HB cells transfected with the indicated amounts of plasmids was detected by CCK-8 (B-C) and colony formation (D) assays.

**Figure S5:** **SNORA14A reduces intracellular succinate and ROS levels via upregulation of SDHB.** (A) The ROS levels of HB cells transfected with OE-NC, OE-SNORA14A or OE-SDHB plasmids were detected by flow cytometry assays. (B) Succinate concentrations in HB/LV-NC and HB/LV-SNORA14A cells transfected with siNC or siSDHB-1/-2 were measured by colorimetric assays. (C) The ROS levels of HB cells with the indicated transfection condition were detected by flow cytometry assays.

**Figure S6:** **SNORA14A promotes HB cell apoptosis and G2/M phase arrest via upregulation of SDHB.** (A) Apoptosis and necrosis of HB/LV-NC and HB/LV-SNORA14A cells transfected with siNC or siSDHB-1/-2 were detected by flow cytometry assays. (B) The cell cycle of HB/LV-NC and HB/LV-SNORA14A cells transfected with siNC or siSDHB-1/-2 was detected by flow cytometry assays.

**Figure S7:** **SNORA14A promotes HB cell apoptosis and G2/M phase arrest via regulation of succinate metabolism.** (A) Apoptosis and necrosis of HB/LV-NC and HB/LV-SNORA14A cells supplemented with succinate were detected by flow cytometry assays. (B) The cell cycle of HB/LV-NC and HB/LV-SNORA14A cells supplemented with succinate was detected by flow cytometry assays.

**Figure S8:** ROC curve indicating the value of SNORA14A as a diagnostic biomarker in HB.
