# Supplementary figures and images for: SNORA14A inhibits hepatoblastoma cell proliferation by regulating SDHB-mediated succinate metabolism
Source: Cell Death Discov. 2023 Jan 30;9:36. doi: 10.1038/s41420-023-01325-0 (PMC9886955; doi:10.1038/s41420-023-01325-0)

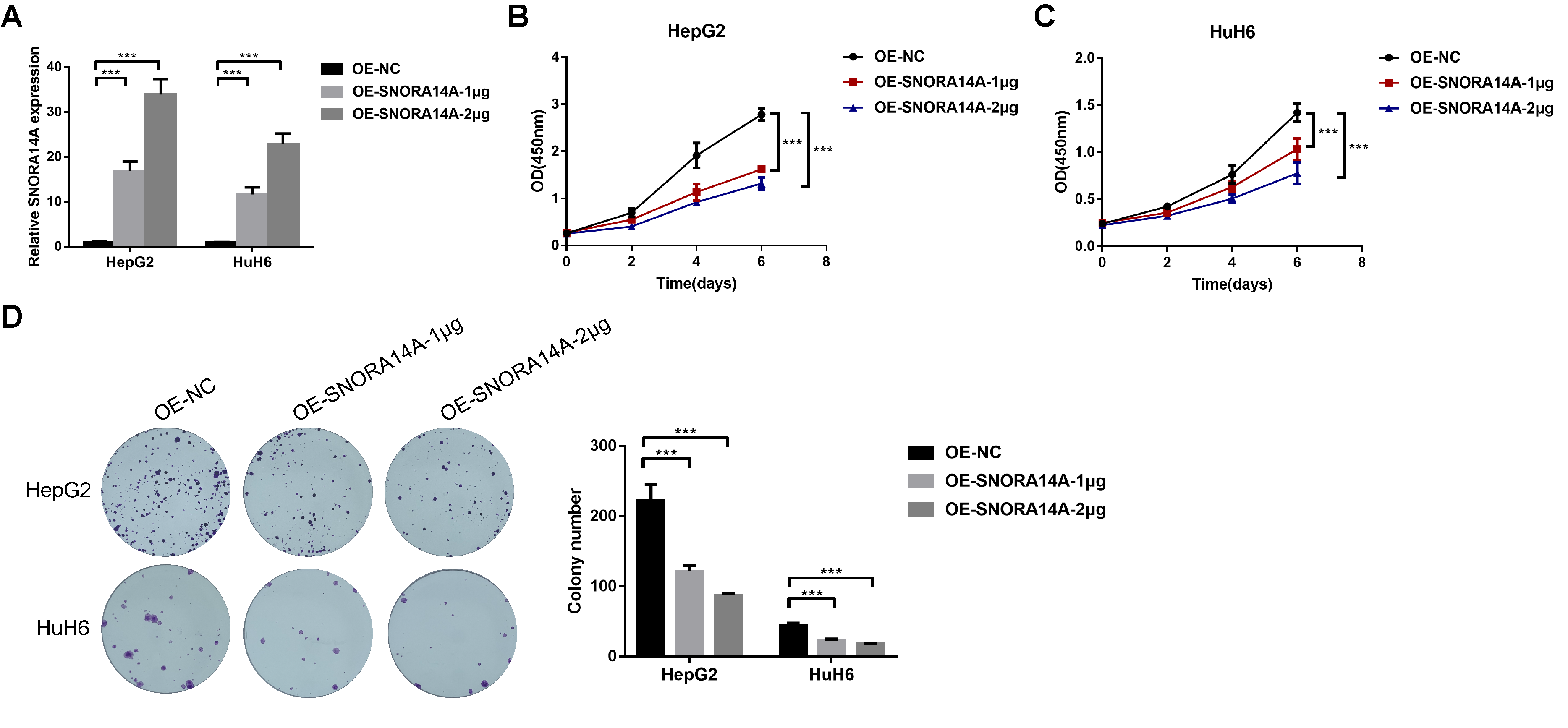

Supplement: Supplementary file 3 — Figure S1 [file 41420_2023_1325_MOESM3_ESM.tif]

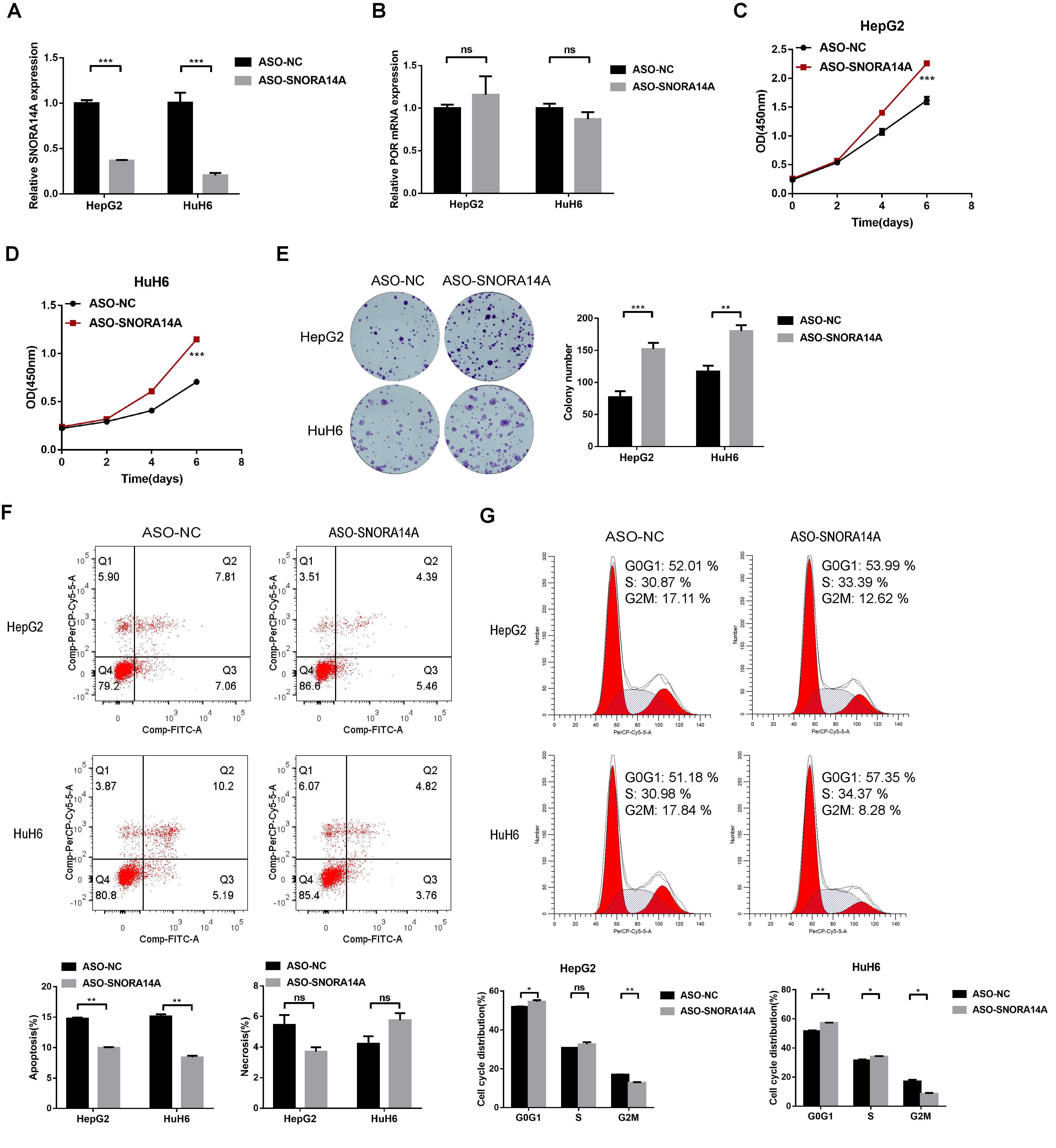

Supplement: Supplementary file 4 — Figure S2 [file 41420_2023_1325_MOESM4_ESM.tif]

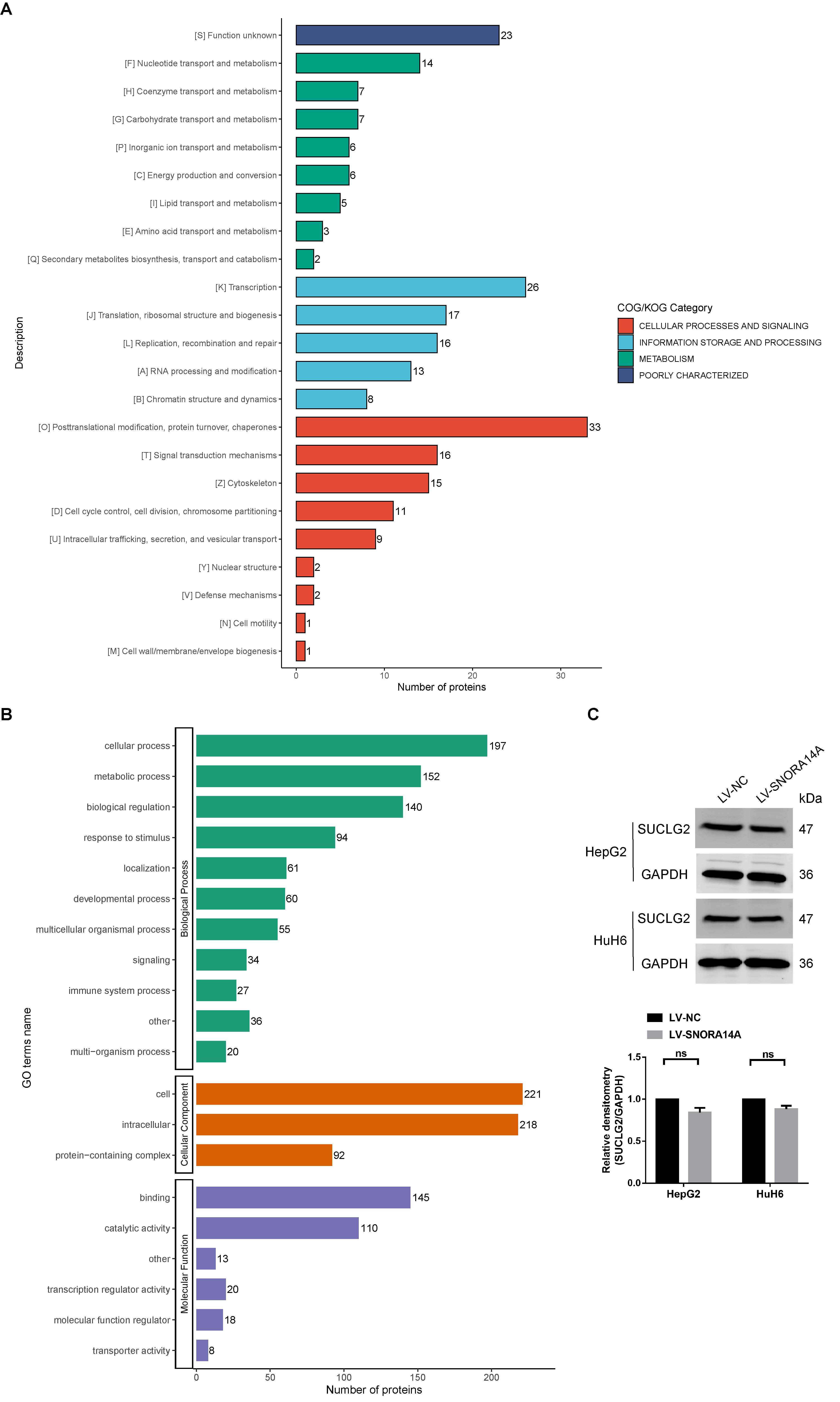

Supplement: Supplementary file 5 — Figure S3 [file 41420_2023_1325_MOESM5_ESM.tif]

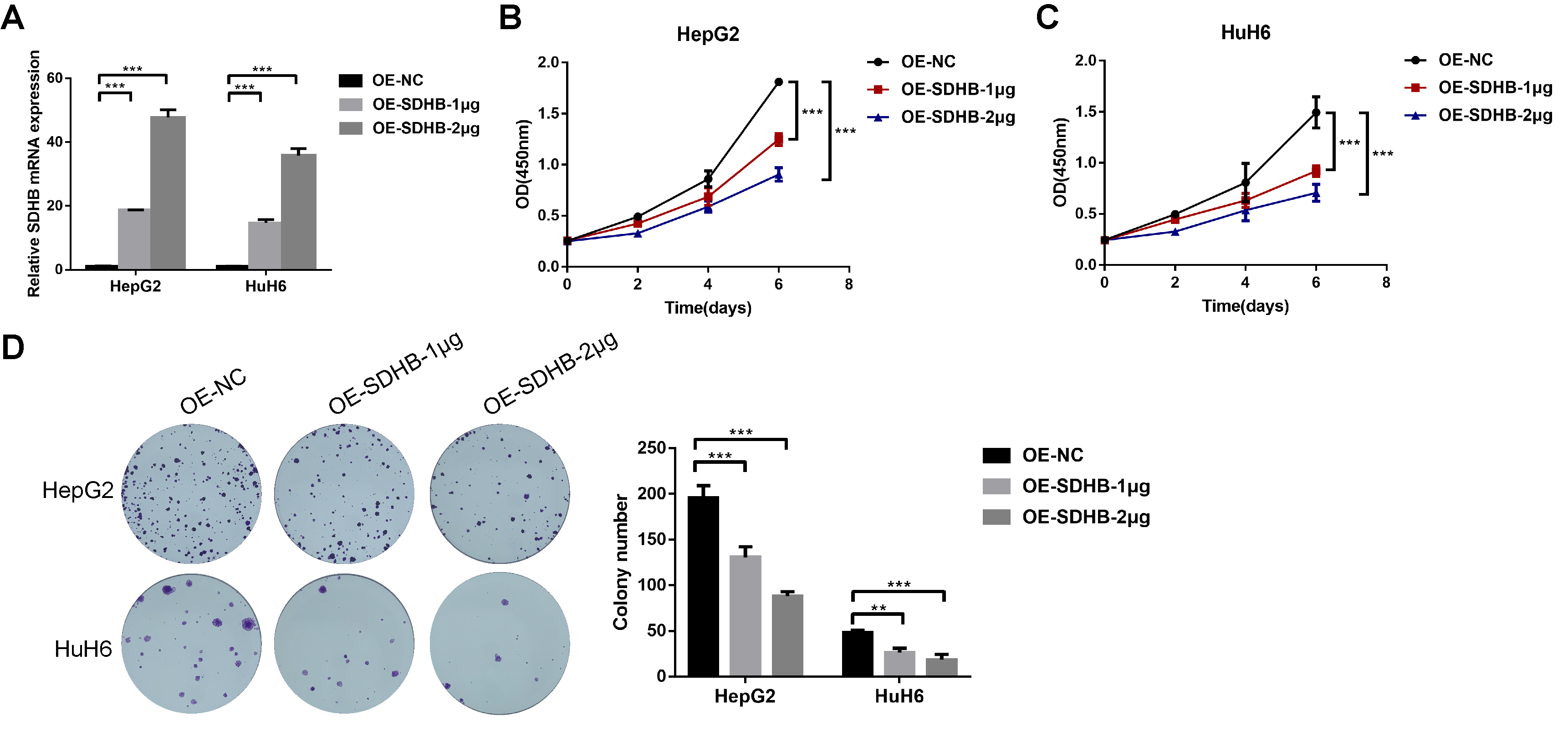

Supplement: Supplementary file 6 — Figure S4 [file 41420_2023_1325_MOESM6_ESM.tif]

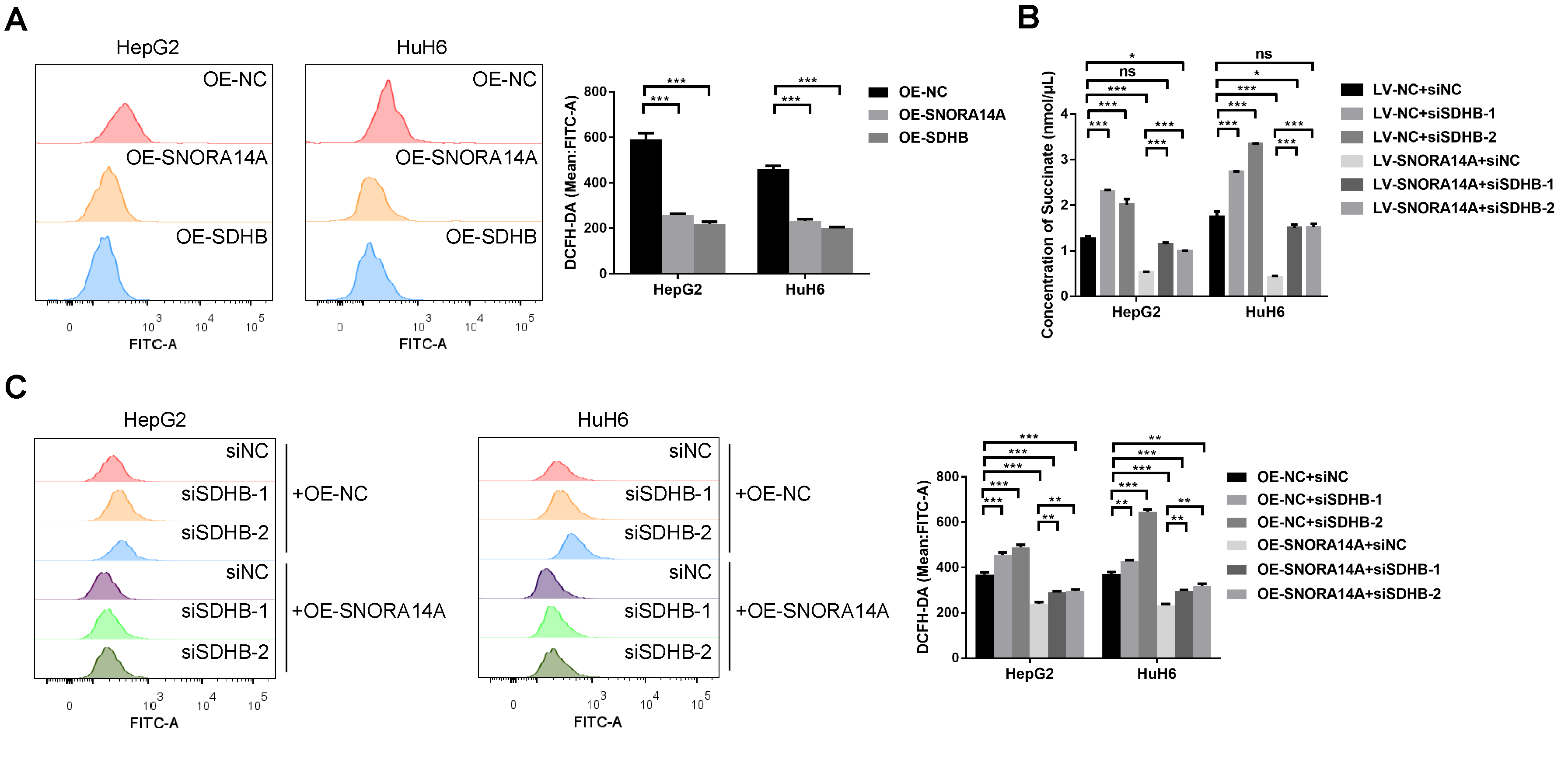

Supplement: Supplementary file 7 — Figure S5 [file 41420_2023_1325_MOESM7_ESM.tif]

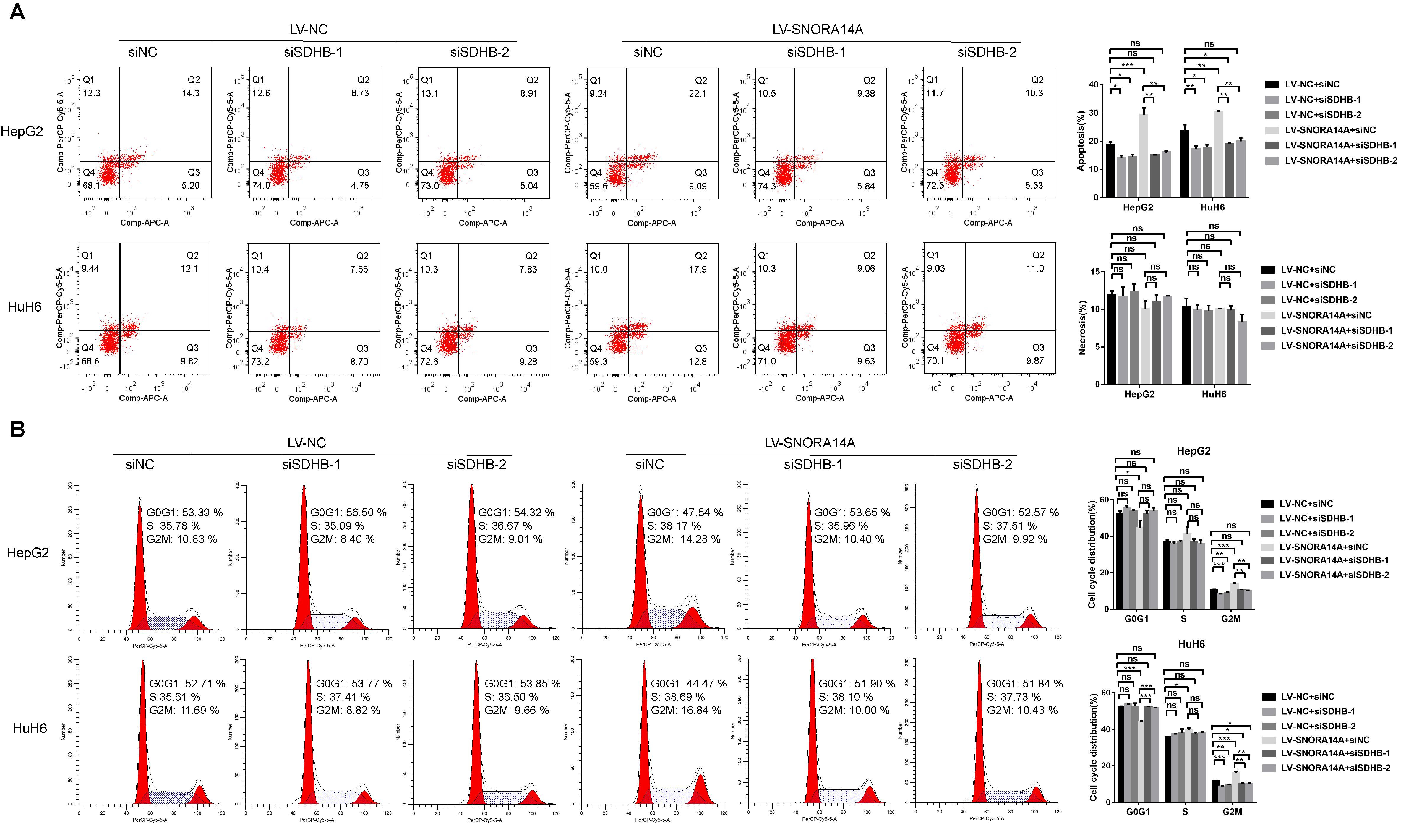

Supplement: Supplementary file 8 — Figure S6 [file 41420_2023_1325_MOESM8_ESM.tif]

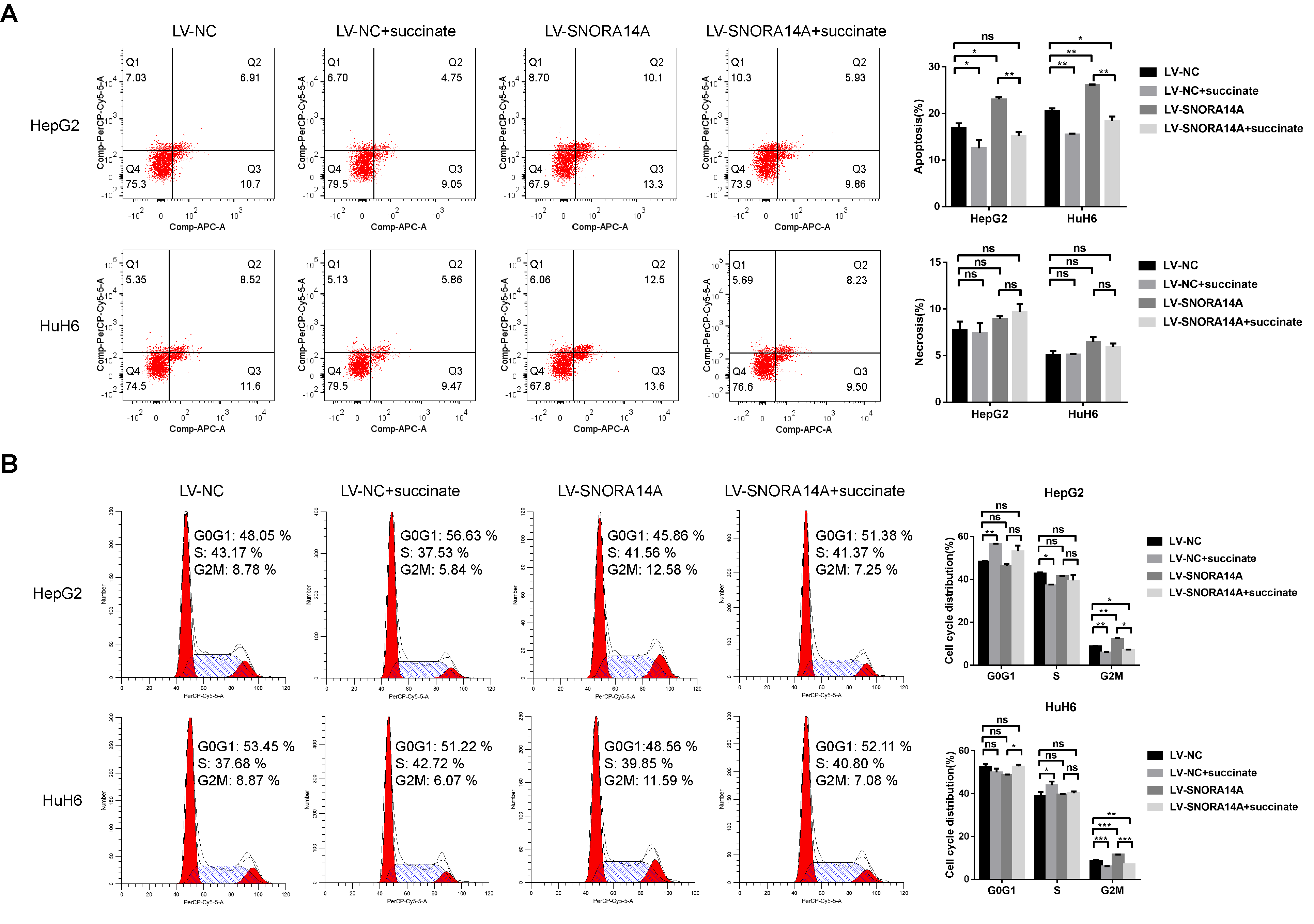

Supplement: Supplementary file 9 — Figure S7 [file 41420_2023_1325_MOESM9_ESM.tif]

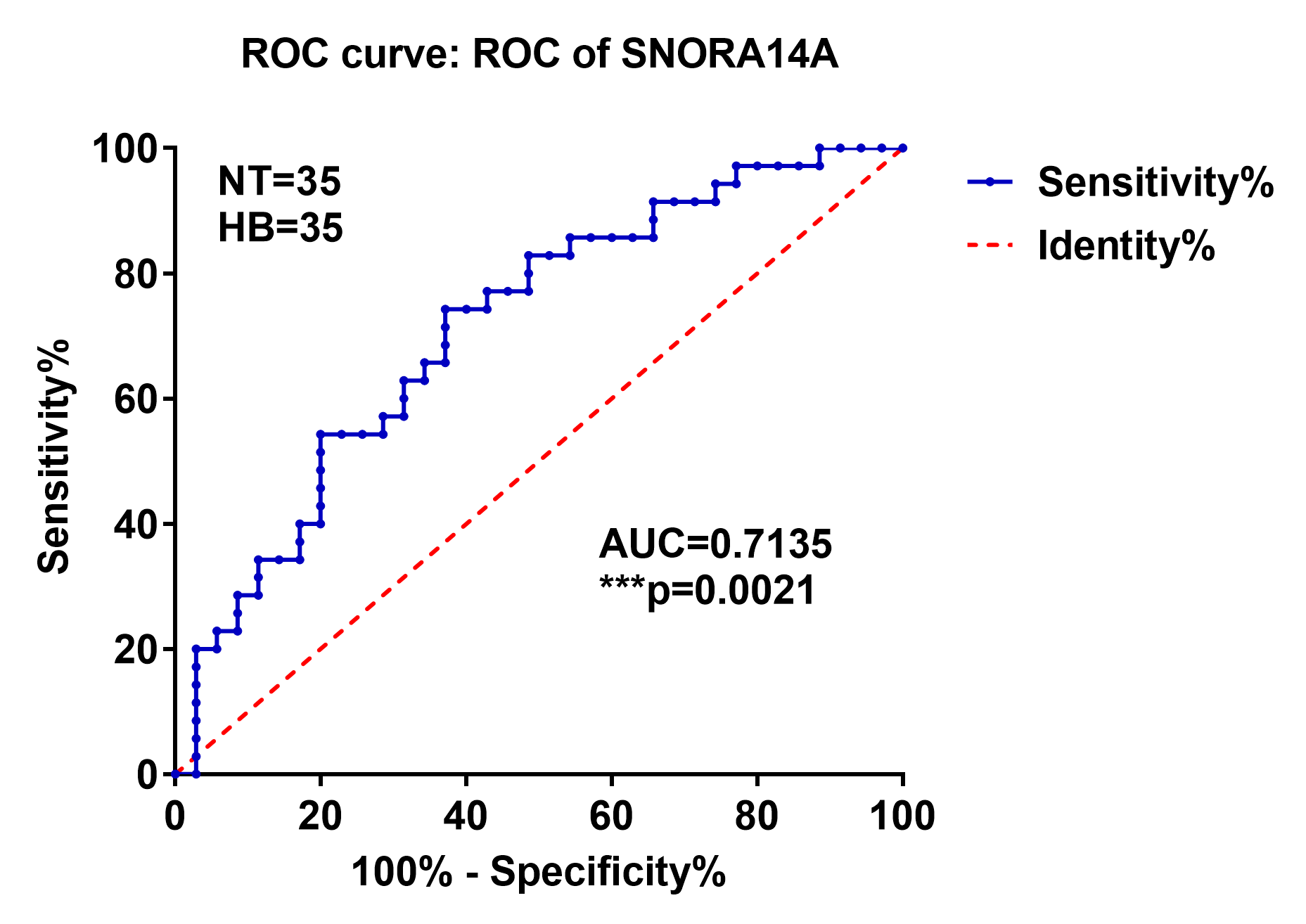

Supplement: Supplementary file 10 — Figure S8 [file 41420_2023_1325_MOESM10_ESM.tif]
