# Supplementary material for: SNORA14A inhibits hepatoblastoma cell proliferation by regulating SDHB-mediated succinate metabolism
Source: Cell Death Discov. 2023 Jan 30;9:36. doi: 10.1038/s41420-023-01325-0 (PMC9886955; doi:10.1038/s41420-023-01325-0)
Supplement: Supplementary file 14 — Table S4 [file 41420_2023_1325_MOESM14_ESM.docx]

**Table S4: The clinical information and pathological characteristics of 35 HB patients.**

| **Variable** | **n** |
| --- | --- |
| **Age at Diagnosis (month)** |  |
| ≥24/<24 | 12/23 |
| **Sex** |  |
| Male/Female | 21/14 |
| **AFP at Diagnosis (ng/ml)** |  |
| ≥1200/<1200 | 30/5 |
| **AFP at Final Detection (ng/ml)** |  |
| ≥5/<5/NA | 23/8/4 |
| **Histology** |  |
| MIX/E/NA | 13/7/15 |
| **PRETEXT** |  |
| I-II/III-IV/NA | 12/16/7 |
| **Tumor Size (cm^3^)** |  |
| ≥500/<500 | 25/10 |
| **Metastasis** |  |
| YES/NO | 9/26 |
