# Supplementary material for: SNORA14A inhibits hepatoblastoma cell proliferation by regulating SDHB-mediated succinate metabolism
Source: Cell Death Discov. 2023 Jan 30;9:36. doi: 10.1038/s41420-023-01325-0 (PMC9886955; doi:10.1038/s41420-023-01325-0)
Supplement: Supplementary file 16 — Table S6 [file 41420_2023_1325_MOESM16_ESM.docx]

**Table S6: The correlation analysis of SDHB protein level and clinicopathological characteristics of 14 HB patients.**

| **HB (n=14)** | **SDHB protein expression** | | **p-value** |
| --- | --- | --- | --- |
|  | **Low** | **High** |  |
| **Age at Diagnosis** |  |  |  |
| ≥24 month | 4 | 1 | 1 |
| <24 month | 7 | 2 |  |
| **Sex** |  |  |  |
| Male | 7 | 1 | 0.538 |
| Female | 4 | 2 |  |
| **AFP at Diagnosis** |  |  |  |
| ≥1200 ng/ml | 10 | 3 | 1 |
| <1200 ng/ml | 1 | 0 |  |
| **AFP at Final Detection** |  |  |  |
| ≥5 ng/ml | 9 | 3 | 1 |
| <5 ng/ml | 1 | 0 |  |
| NA | 1 | 0 |  |
| **Histology** |  |  |  |
| MIX | 5 | 1 | 0.547 |
| E | 3 | 0 |  |
| NA | 3 | 2 |  |
| **PRETEXT** |  |  |  |
| I-II | 1 | 3 | 0.011 |
| III-IV | 9 | 0 |  |
| NA | 1 | 0 |  |
| **Tumor Size** |  |  |  |
| ≥500 cm^3^ | 8 | 2 | 1 |
| <500 cm^3^ | 3 | 1 |  |
| **Metastasis** |  |  |  |
| YES | 9 | 0 | 0.027 |
| NO | 2 | 3 |  |
| **SNORA14A Expression** |  |  |  |
| Low | 10 | 0 | 0.011 |
| High | 1 | 3 |  |
