# Supplementary material for: SNORA14A inhibits hepatoblastoma cell proliferation by regulating SDHB-mediated succinate metabolism
Source: Cell Death Discov. 2023 Jan 30;9:36. doi: 10.1038/s41420-023-01325-0 (PMC9886955; doi:10.1038/s41420-023-01325-0)
Supplement: Supplementary file 17 — Original Western blotting images [file 41420_2023_1325_MOESM17_ESM.pdf]

Original images

Figure 3G

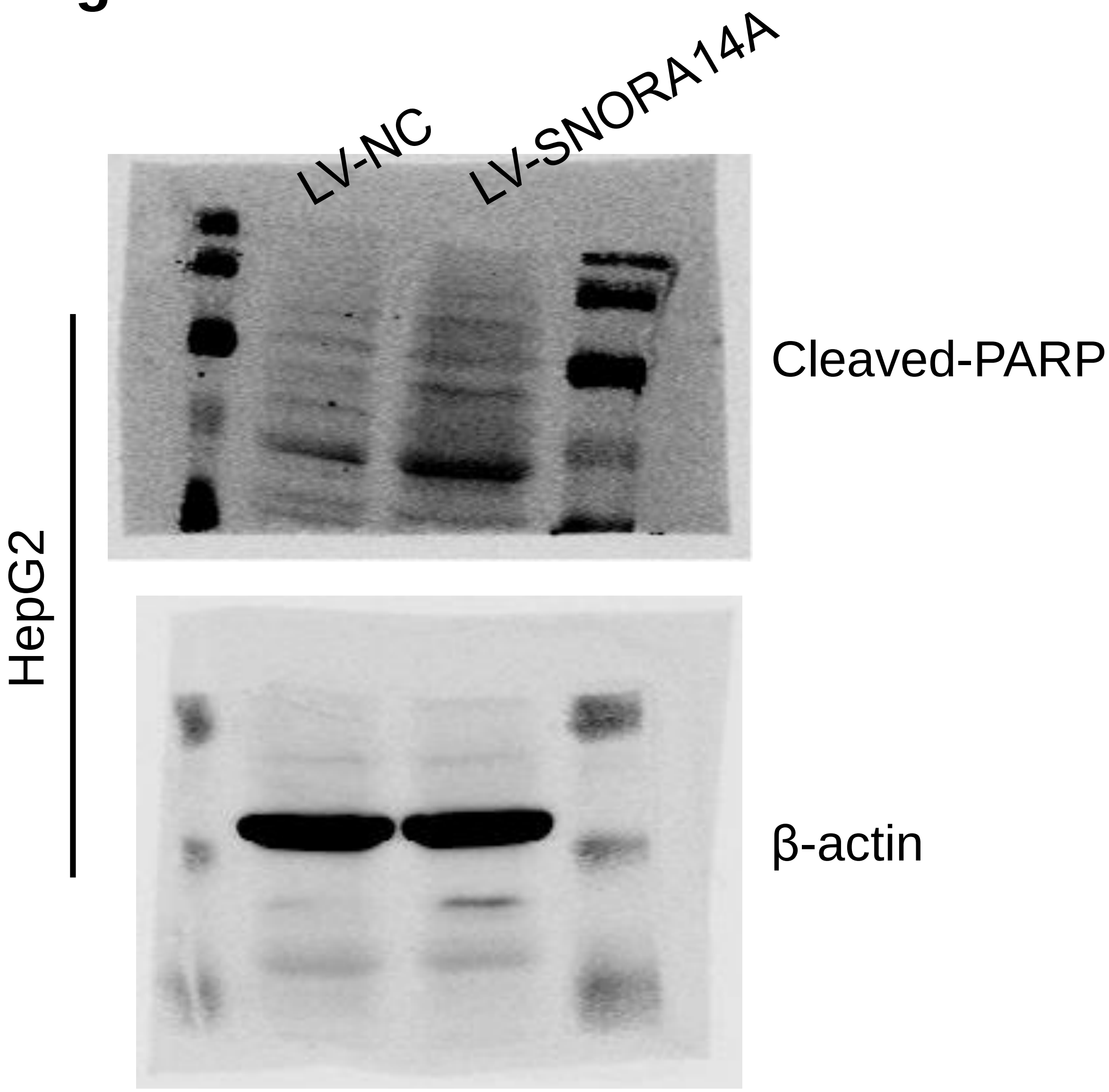

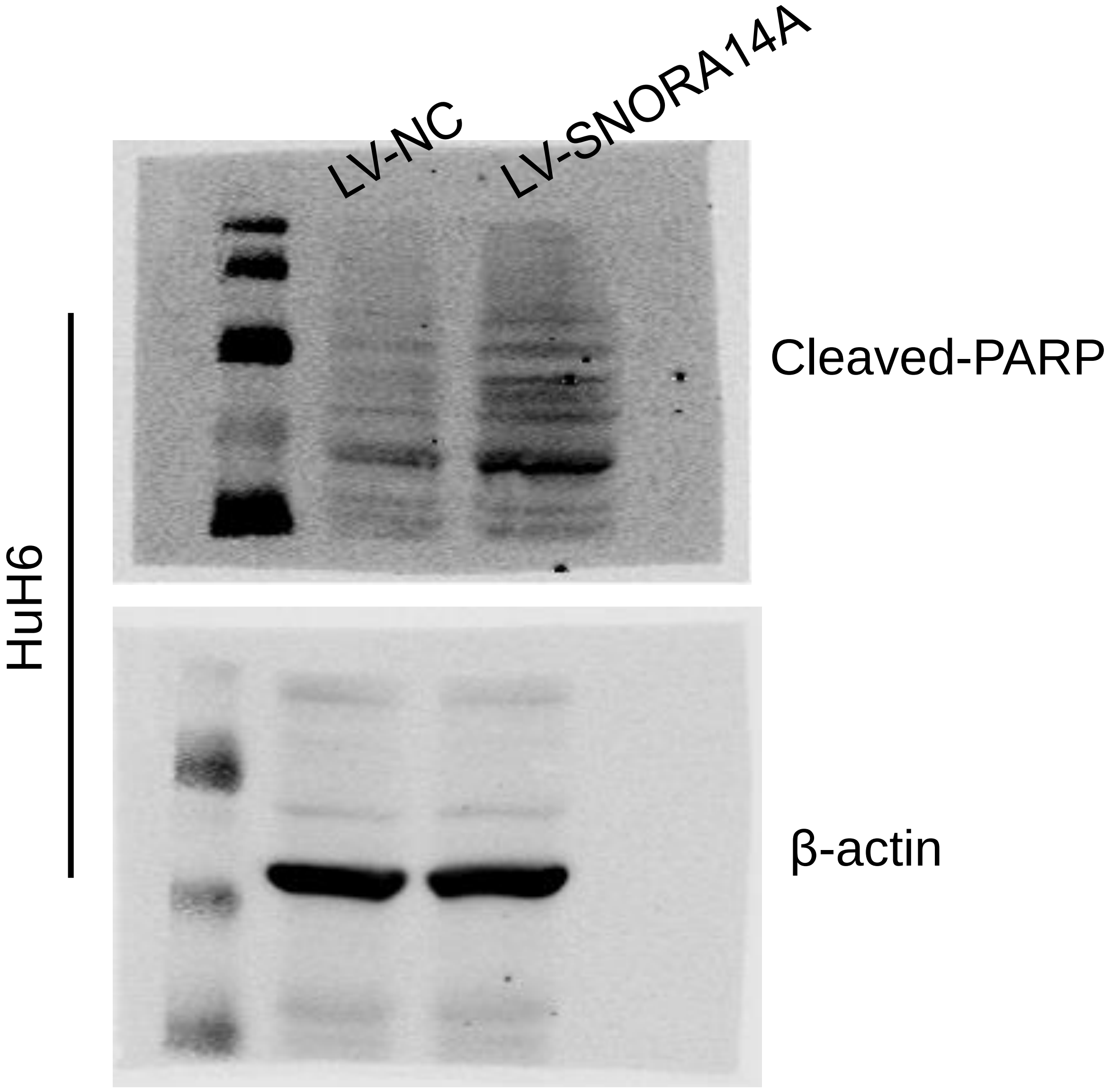

Figure 5E

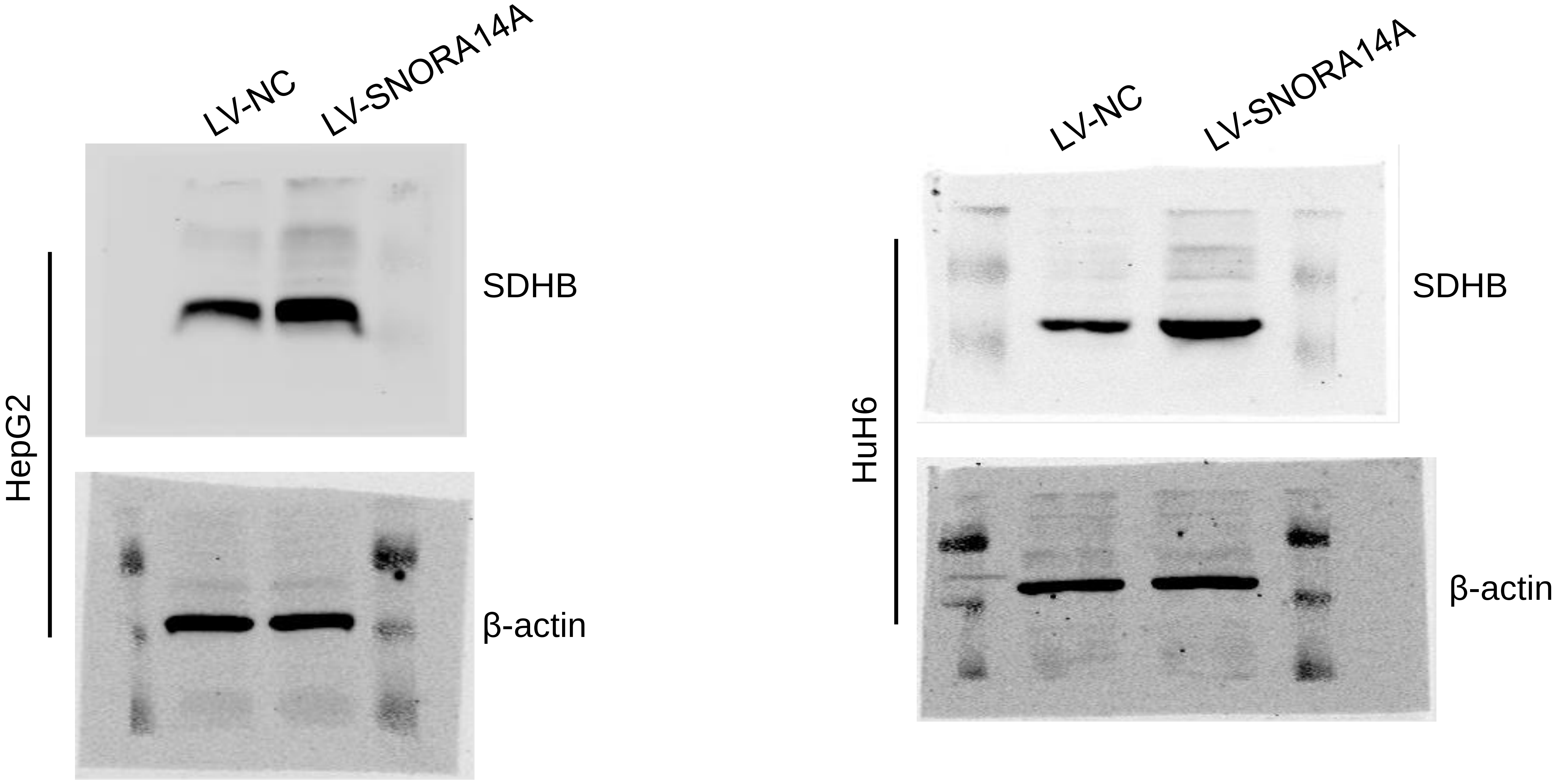
LV-NC
LV-SNORA14A
SDHB
β-actin
HepG2
LV-NC
LV-SNORA14A
SDHB
β-actin
HuH6

Figure 5F

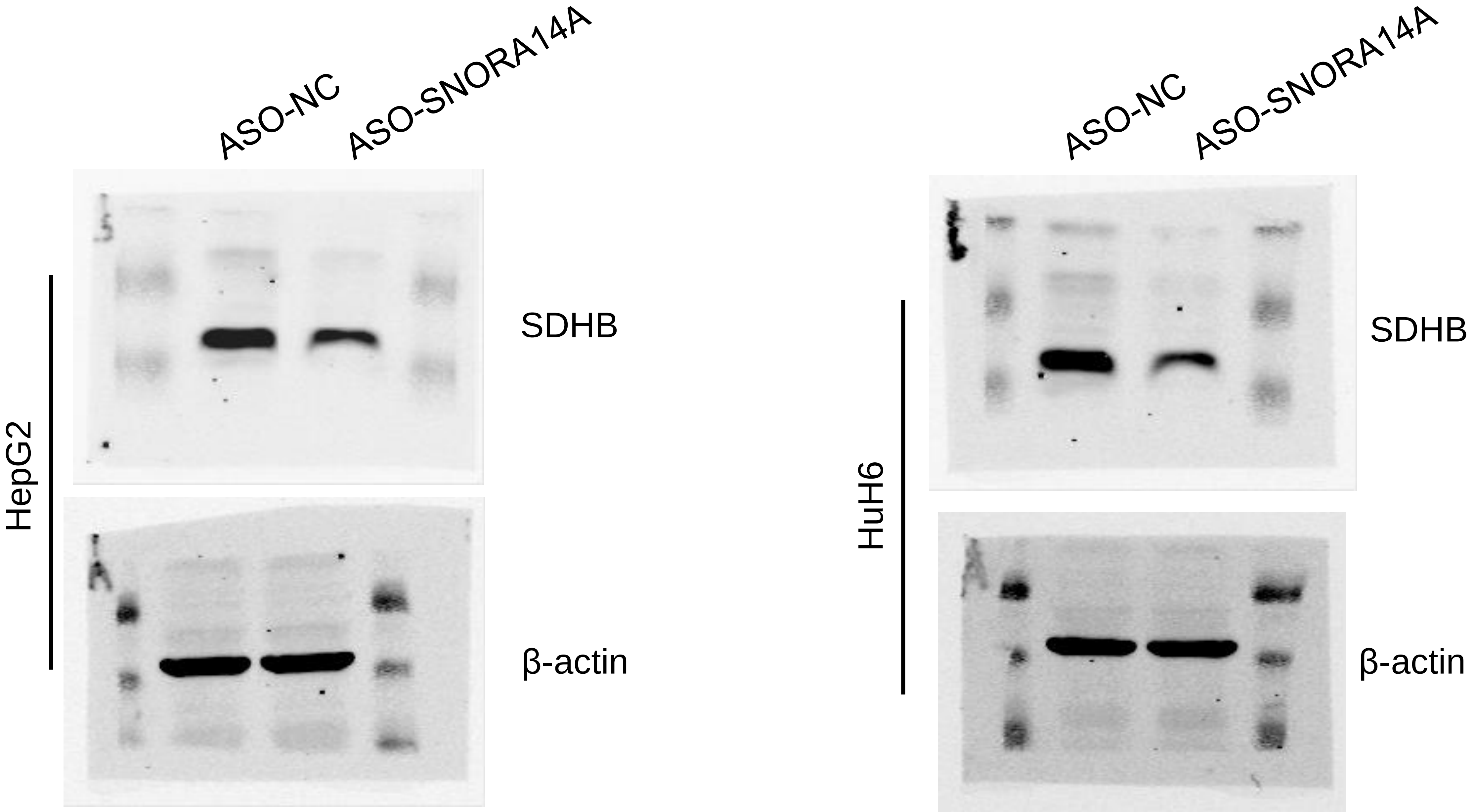
ASO-NC
ASO-SNORA14A
SDHB
β-actin
HepG2
ASO-NC
ASO-SNORA14A
SDHB
β-actin
HuH6

Figure 5G

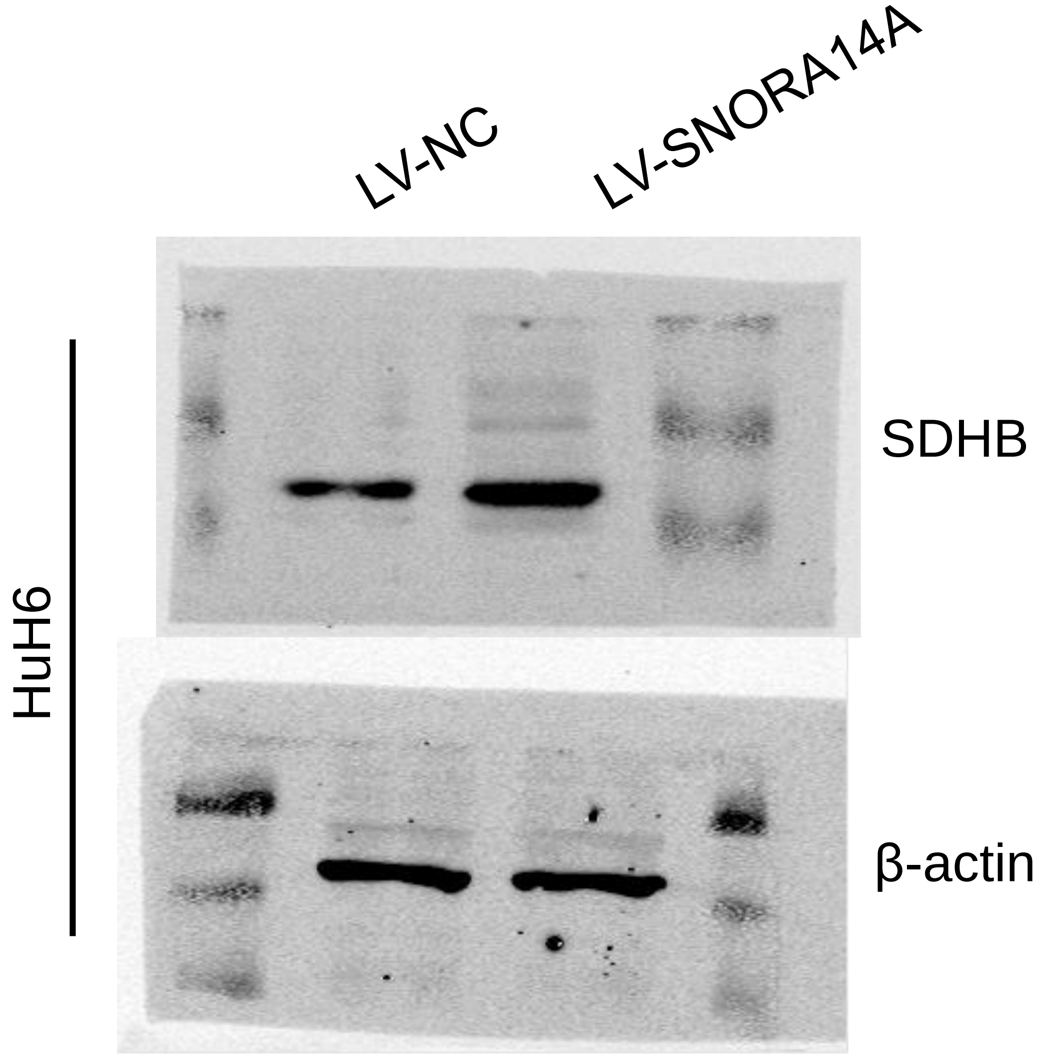

Figure 5K

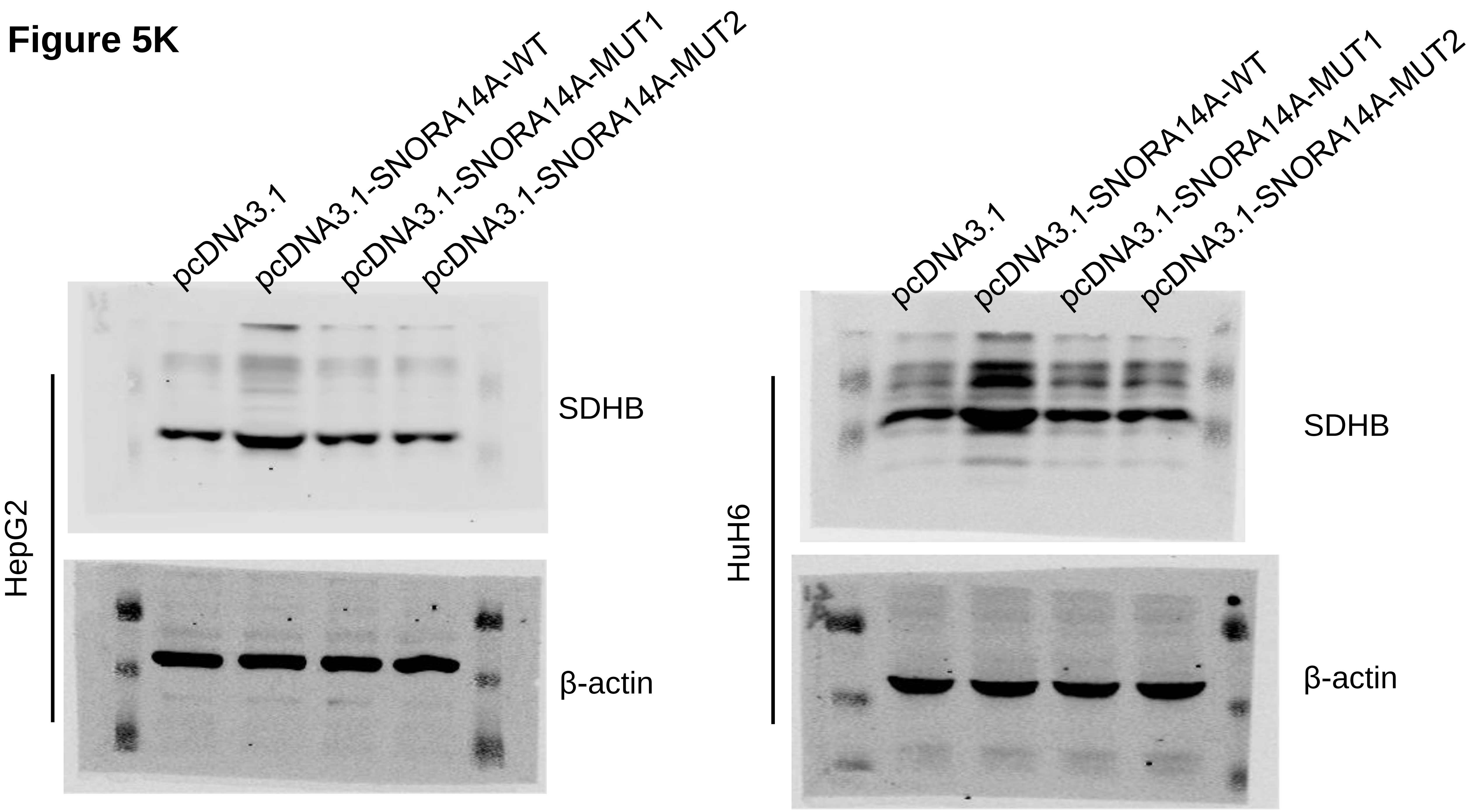
pcDNA3.1
pcDNA3.1-SNORA14A-WT
pcDNA3.1-SNORA14A-MUT1
pcDNA3.1-SNORA14A-MUT2
SDHB
β-actin
HepG2
pcDNA3.1
pcDNA3.1-SNORA14A-WT
pcDNA3.1-SNORA14A-MUT1
pcDNA3.1-SNORA14A-MUT2
SDHB
β-actin
HuH6

Figure 6D

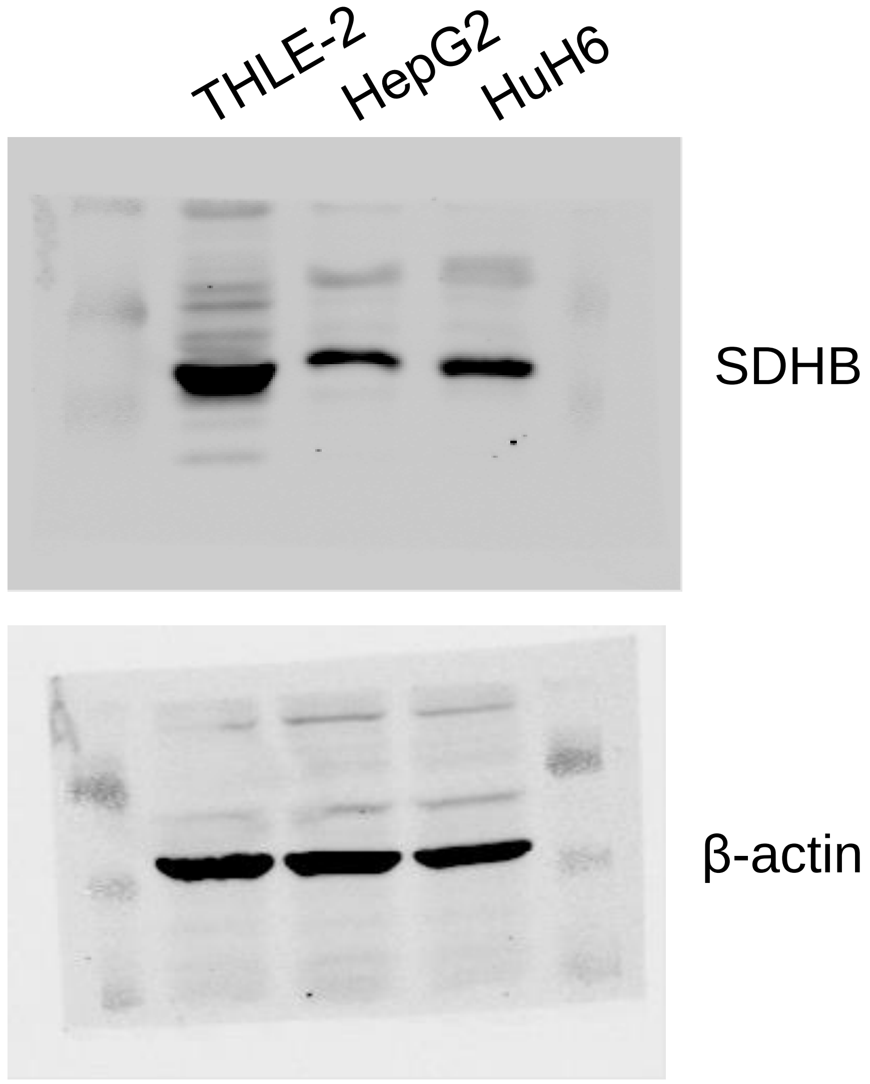

Figure 6E

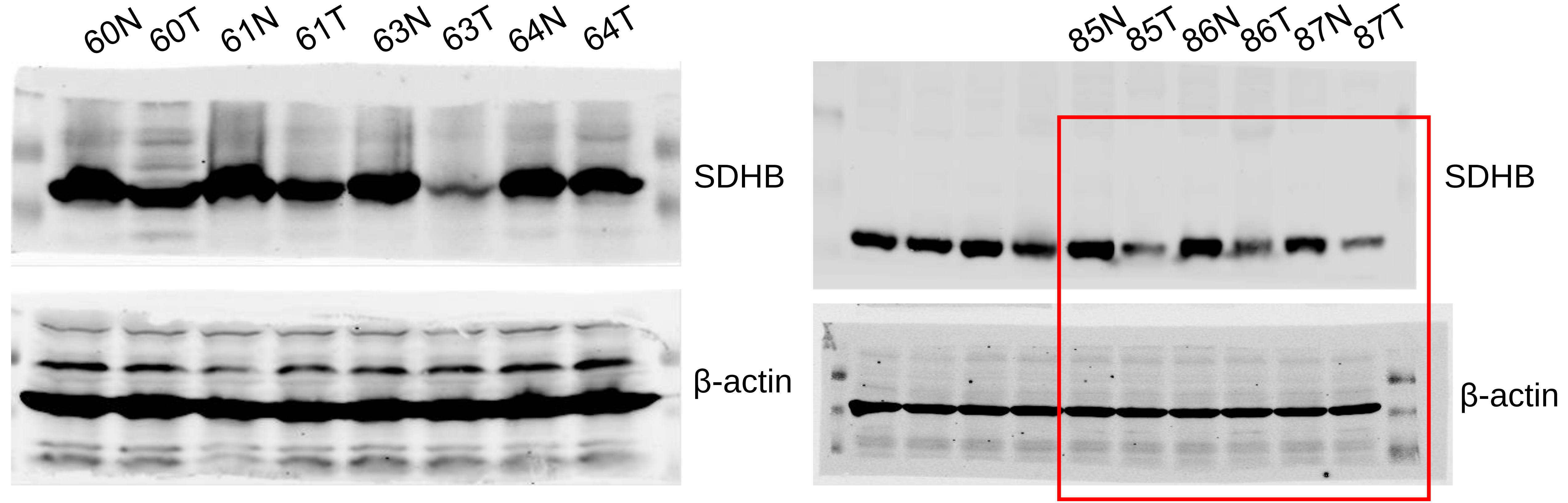

60N
60T
61N
61T
63N
63T
64N
64T
SDHB
β-actin
85N
85T
86N
86T
87N
87T
SDHB
β-actin

Figure 6I

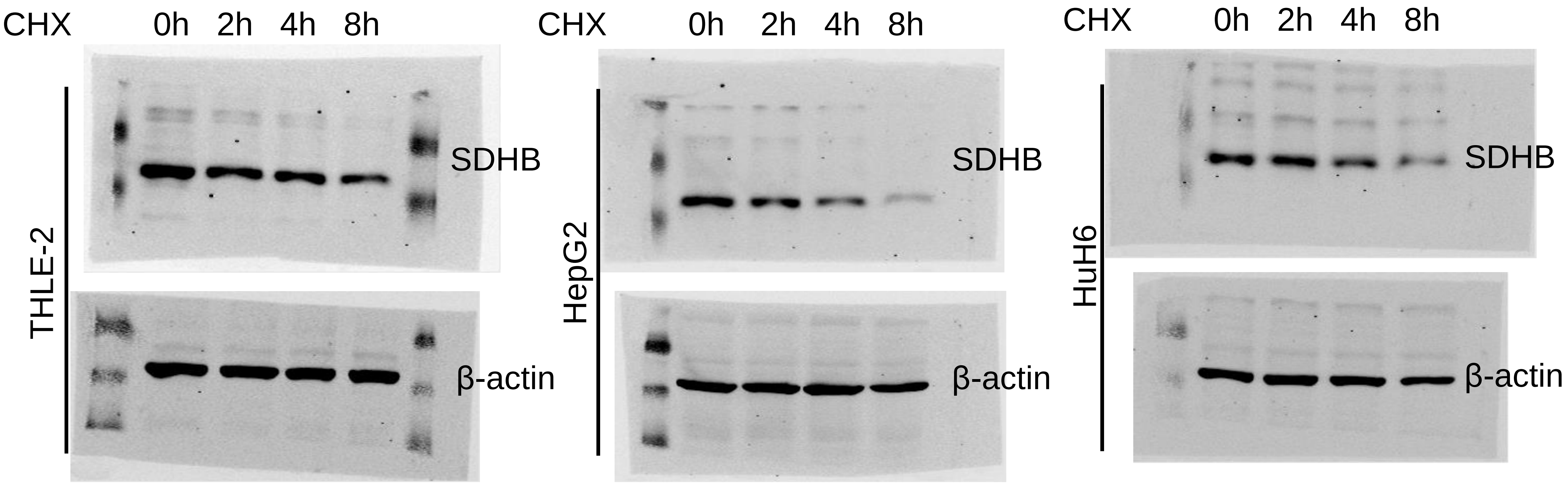
CHX
0h
2h
4h
8h
SDHB
β-actin
THLE-2
CHX
0h
2h
4h
8h
SDHB
β-actin
HepG2
CHX
0h
2h
4h
8h
SDHB
β-actin
HuH6

Figure 7B

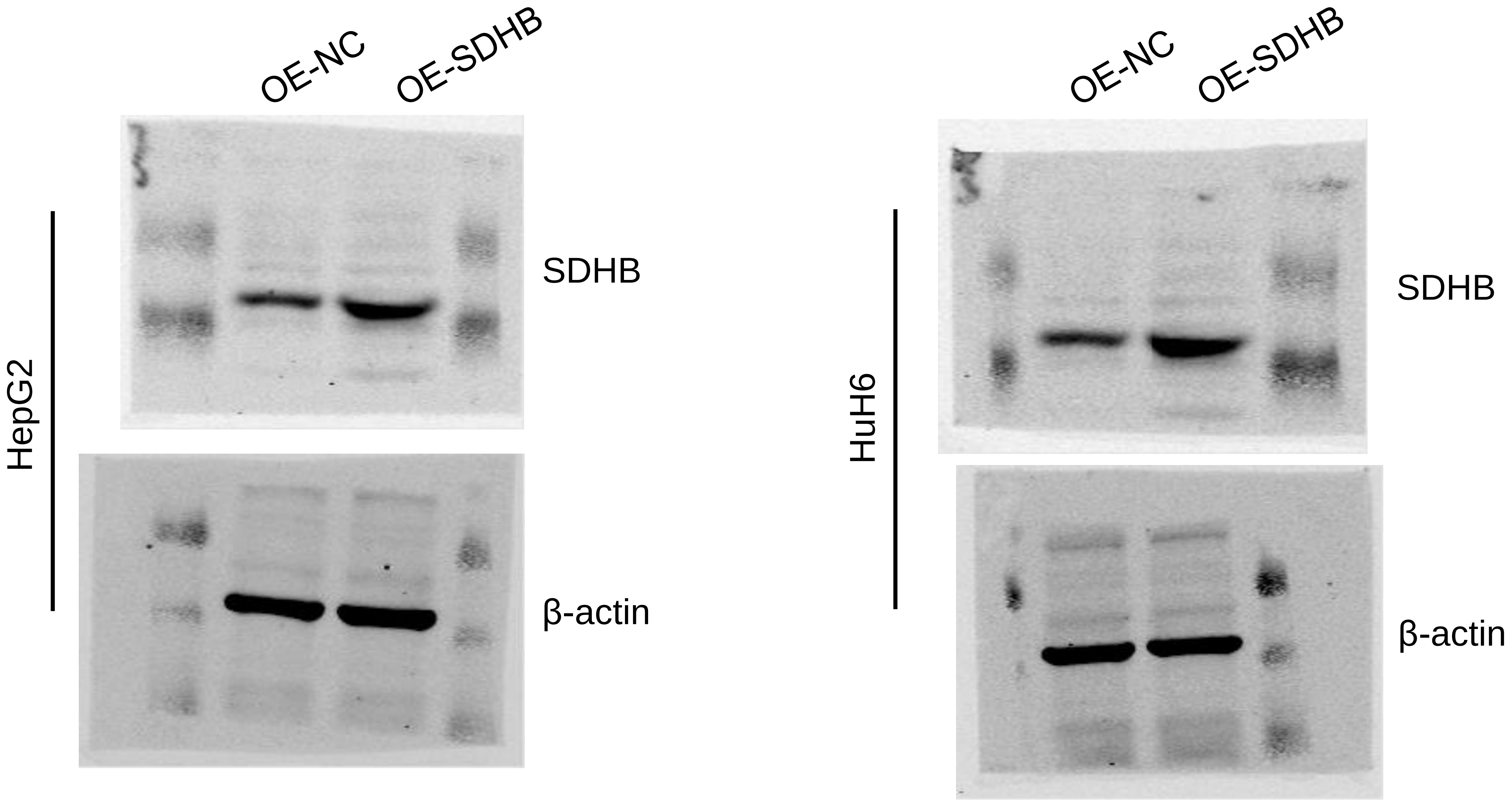

Figure 7G

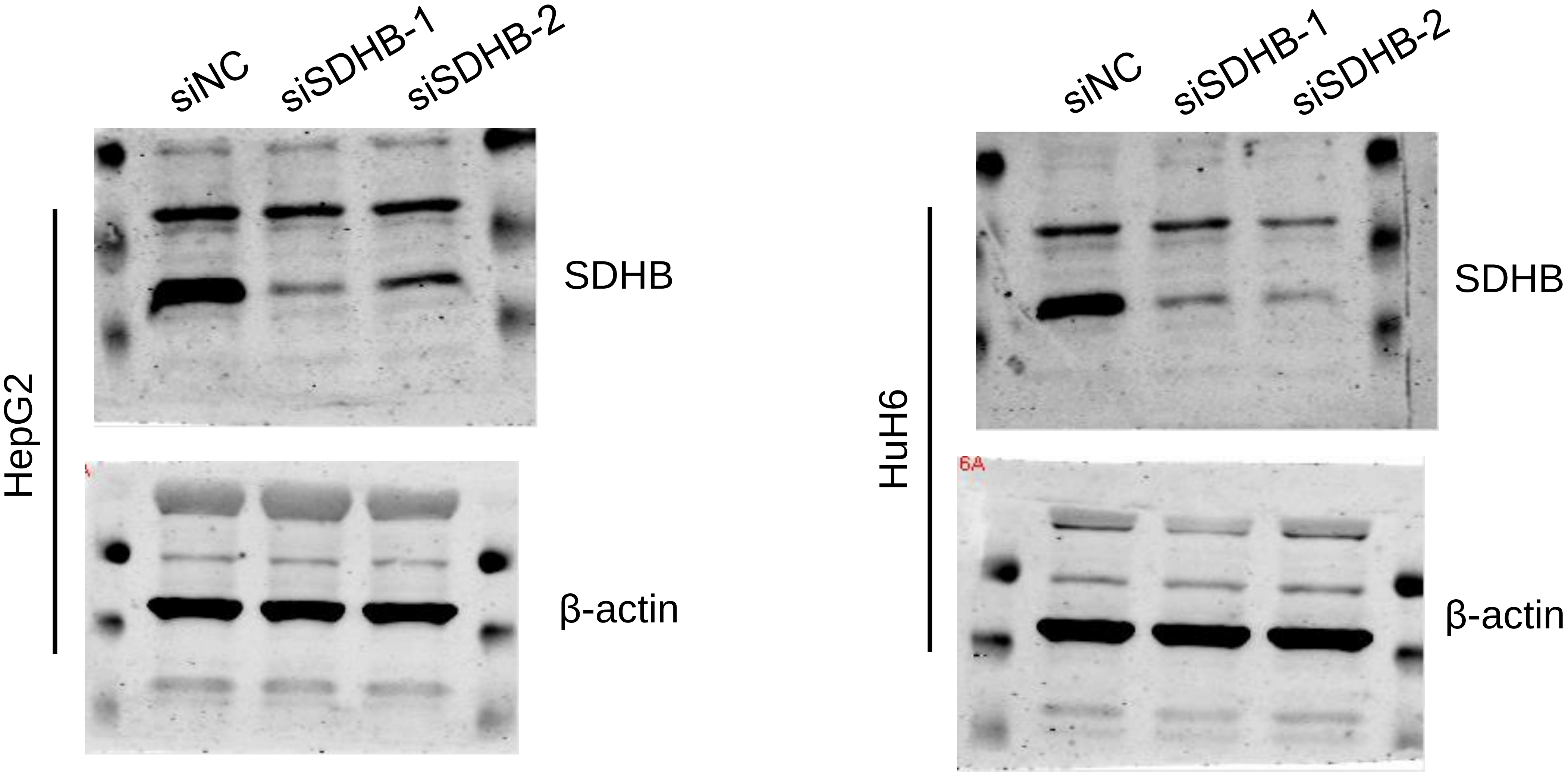
siNC
siSDHB-1
siSDHB-2
SDHB
β-actin
HepG2
siNC
siSDHB-1
siSDHB-2
SDHB
β-actin
HuH6

Figure 7K

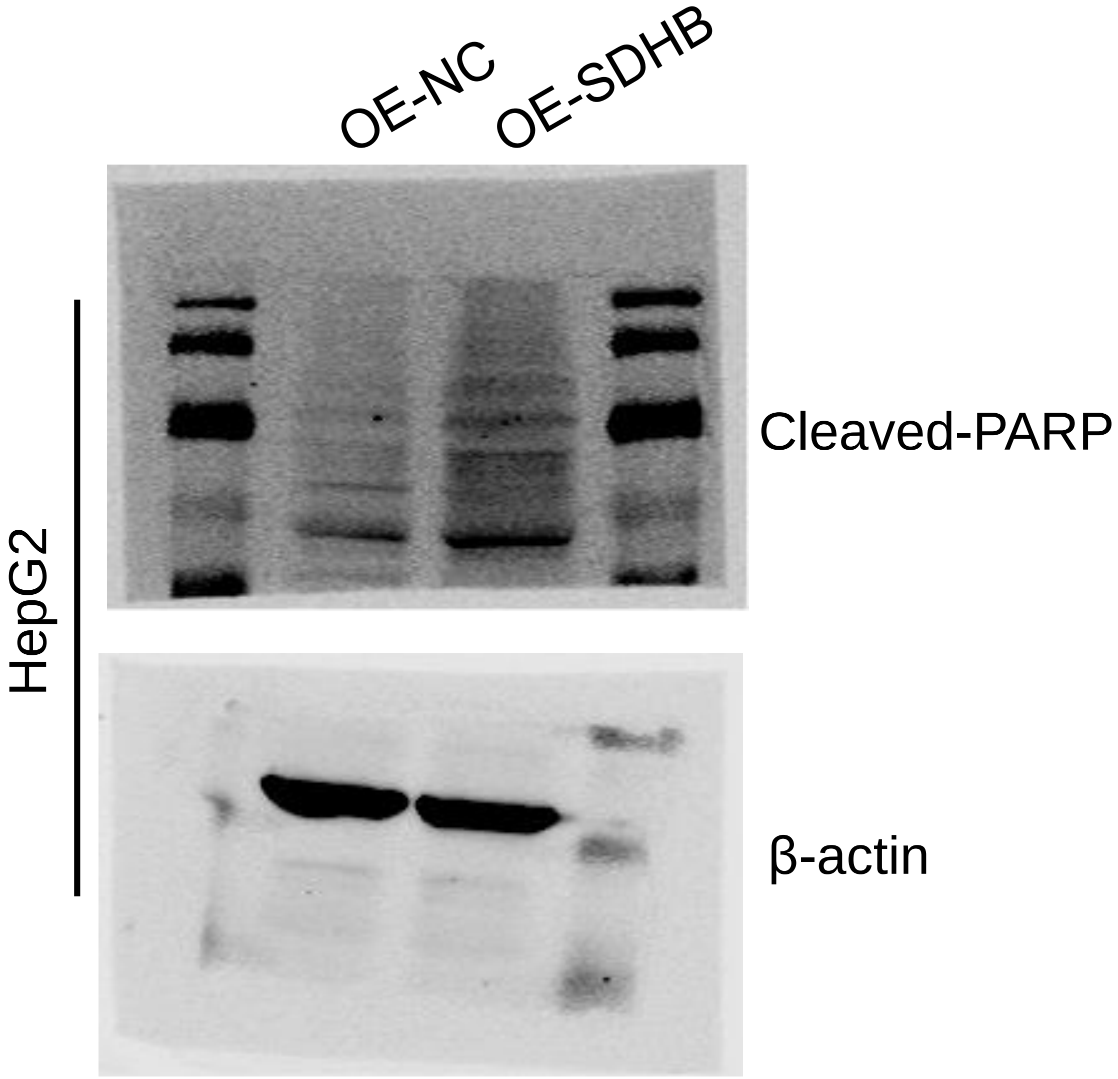
OE-NC
OE-SDHB
Cleaved-PARP
β-actin
HepG2

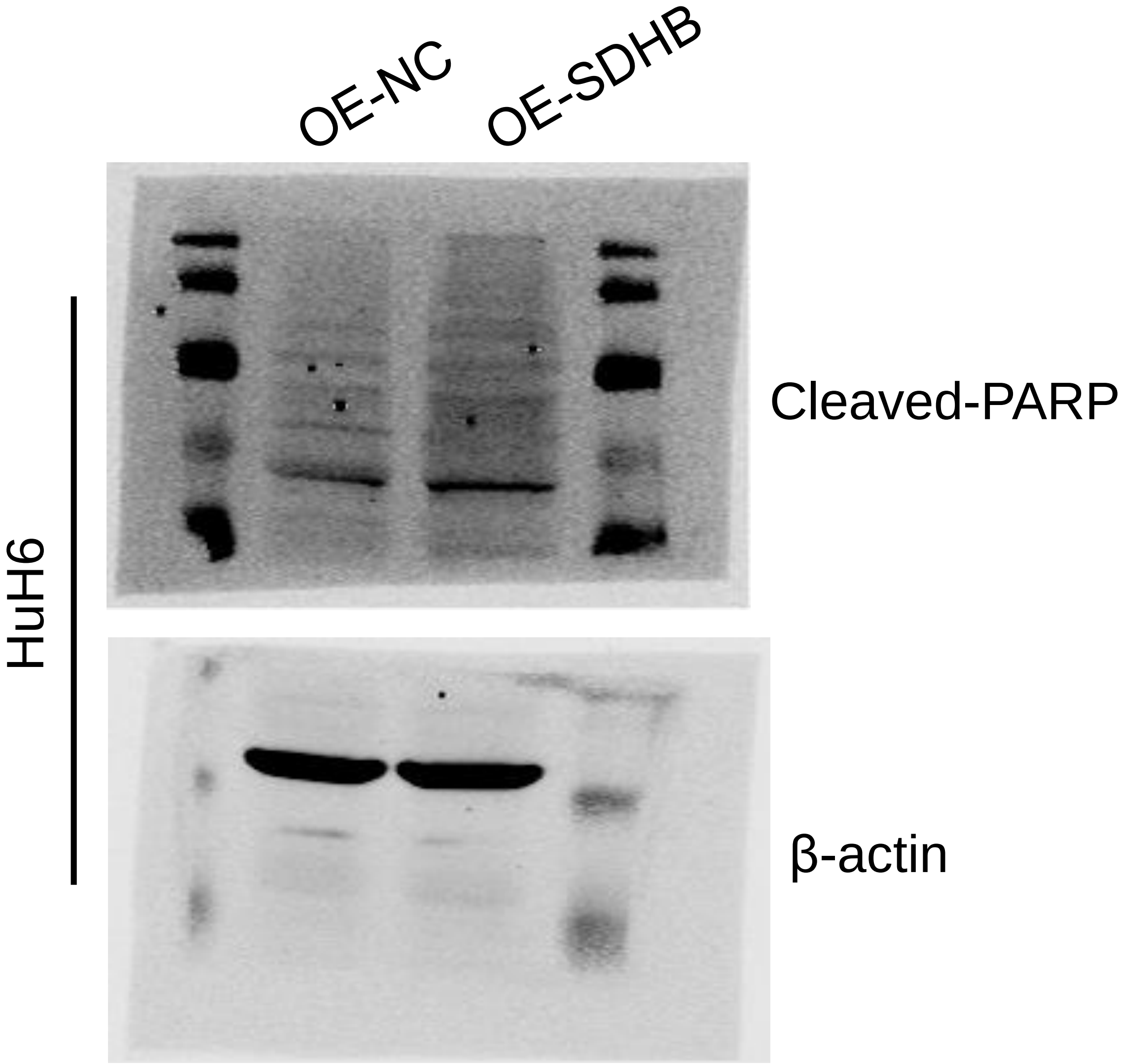
OE-NC
OE-SDHB
Cleaved-PARP
β-actin
HuH6

Figure 8K

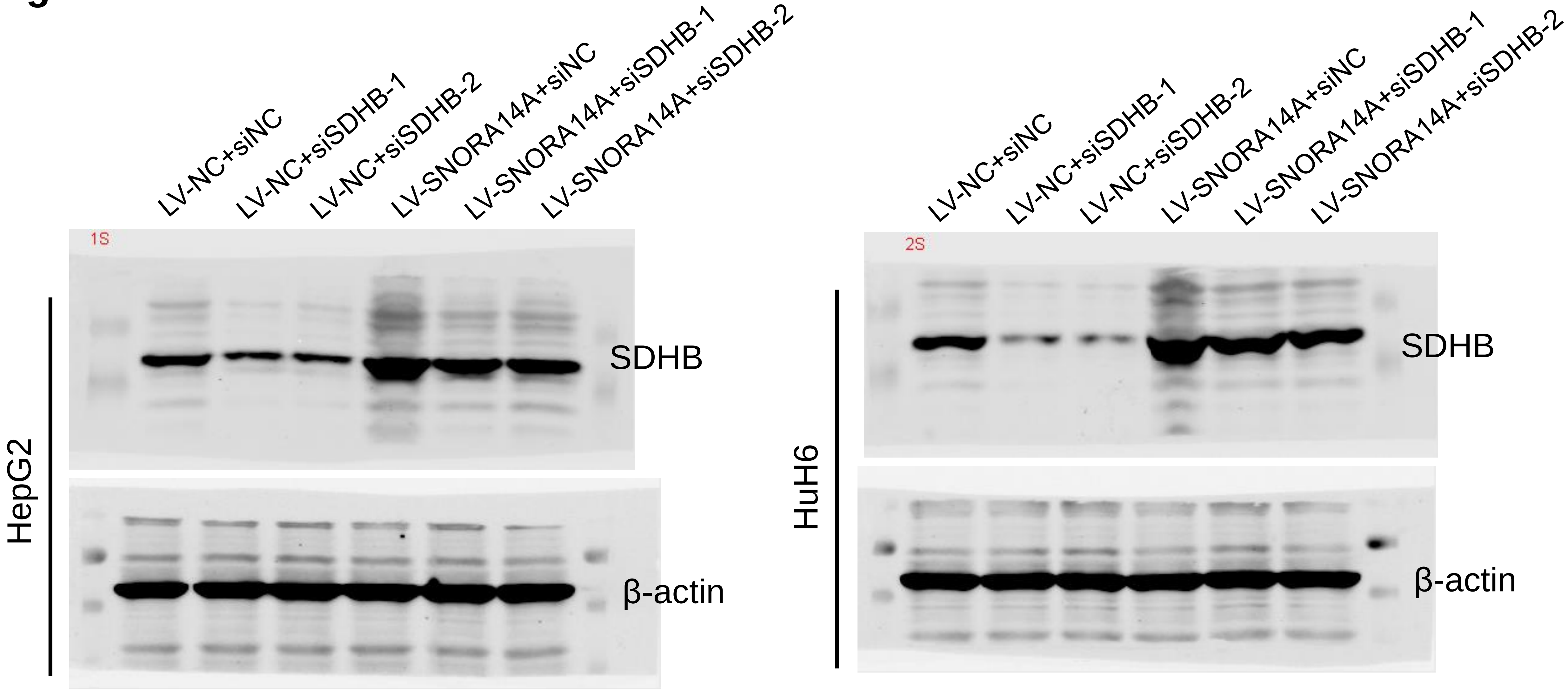
LV-NC+siNC
LV-NC+siSDHB-1
LV-NC+siSDHB-2
LV-SNORA14A+siNC
LV-SNORA14A+siSDHB-1
LV-SNORA14A+siSDHB-2
1S
SDHB
β-actin
HepG2
LV-NC+siNC
LV-NC+siSDHB-1
LV-NC+siSDHB-2
LV-SNORA14A+siNC
LV-SNORA14A+siSDHB-1
LV-SNORA14A+siSDHB-2
2S
SDHB
β-actin
HuH6

Figure S3C

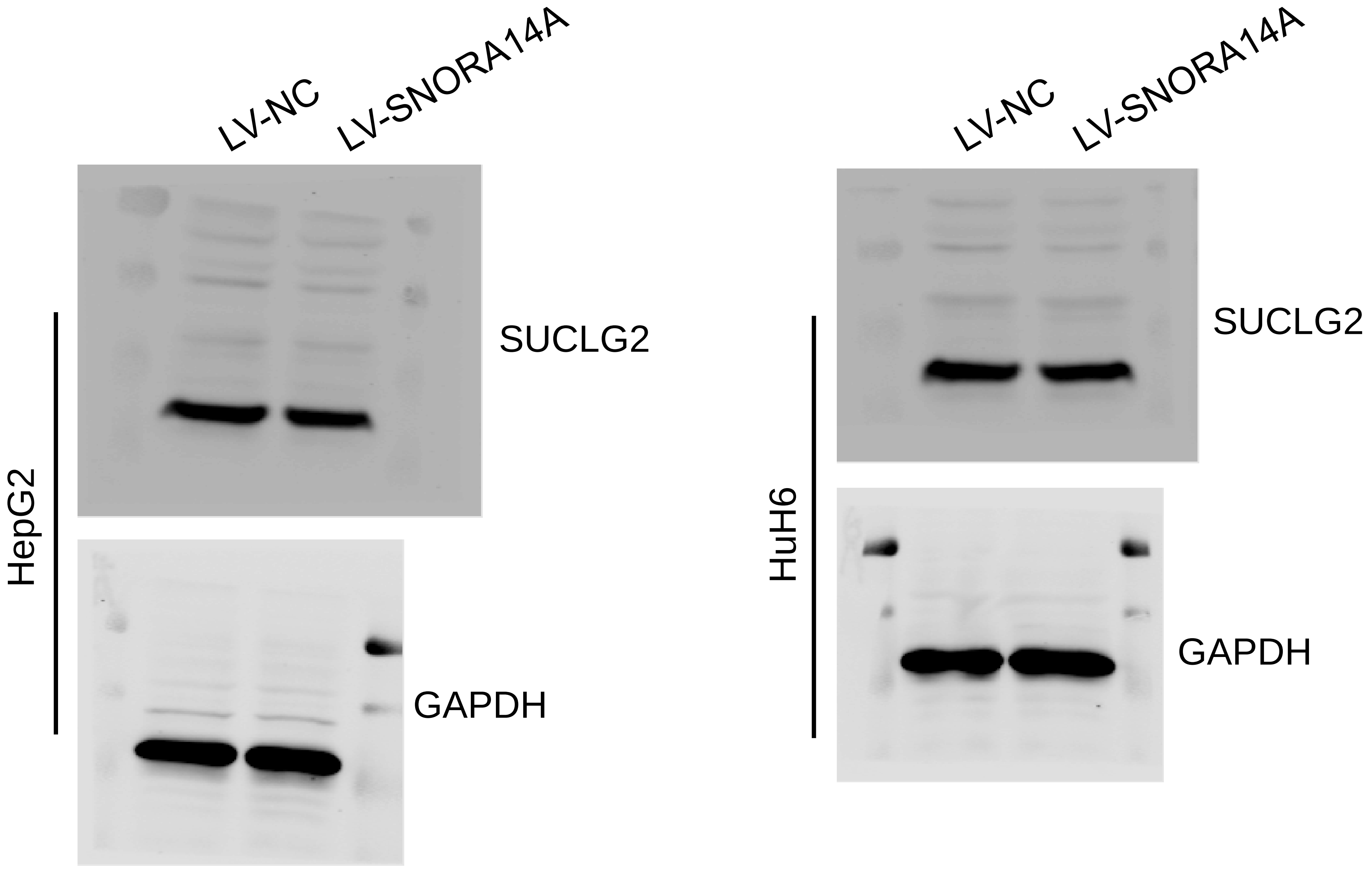
LV-NC
LV-SNORA14A
SUCLG2
GAPDH
HepG2
LV-NC
LV-SNORA14A
SUCLG2
GAPDH
HuH6
